# Suggested deafness during hypnosis and simulation of hypnosis compared to a distraction and control condition: A study on subjective experience and cortical brain responses

**DOI:** 10.1371/journal.pone.0240832

**Published:** 2020-10-29

**Authors:** Marcel Franz, Barbara Schmidt, Holger Hecht, Ewald Naumann, Wolfgang H. R. Miltner

**Affiliations:** 1 Institute of Psychology, Friedrich Schiller University of Jena, Jena, Germany; 2 Institute of Psychology, University of Trier, Trier, Germany; 3 Medical Faculty of the Friedrich Schiller University of Jena, Jena, Germany; Universita degli Studi di Roma La Sapienza, ITALY

## Abstract

Hypnosis is a powerful tool to affect the processing and perception of stimuli. Here, we investigated the effects of hypnosis on the processing of auditory stimuli, the time course of event-related-potentials (ERP; N1 and P3b amplitudes) and the activity of cortical sources of the P3b component. Forty-eight participants completed an auditory oddball paradigm composed of standard, distractor, and target stimuli during a hypnosis (HYP), a simulation of hypnosis (SIM), a distraction (DIS), and a control (CON) condition. During HYP, participants were suggested that an earplug would obstruct the perception of tones and during SIM they should pretend being hypnotized and obstructed to hear the tones. During DIS, participants’ attention was withdrawn from the tones by focusing participants’ attention onto a film. In each condition, subjects were asked to press a key whenever a target stimulus was presented. Behavioral data show that target hit rates and response time became significantly reduced during HYP and SIM and loudness ratings of tones were only reduced during HYP. Distraction from stimuli by the film was less effective in reducing target hit rate and tone loudness. Although, the N1 amplitude was not affected by the experimental conditions, the P3b amplitude was significantly reduced in HYP and SIM compared to CON and DIS. In addition, source localization results indicate that only a small number of neural sources organize the differences of tone processing between the control condition and the distraction, hypnosis, and simulation of hypnosis conditions. These sources belong to brain areas that control the focus of attention, the discrimination of auditory stimuli, and the organization of behavioral responses to targets. Our data confirm that deafness suggestions significantly change auditory processing and perception but complete deafness is hard to achieve during HYP. Therefore, the term ‘deafness’ may be misleading and should better be replaced by ‘hypoacusis’.

## Introduction

Clinical experience and experimental studies have shown that hypnotic suggestions can significantly modify the perception of our internal and external world. There is clear evidence that people can completely relieve from pain with the help of hypnotically induced hypoalgesic suggestions either during surgical interventions like cataract surgery [1, 2], bone marrow aspirations, lumbar punctures and voiding cystourethograms (for all these examples see: [3]), or dental surgery [4, 5]. Other examples are provided by hypnotized fakirs [6, 7] and Tamil believers [8, 9] in hypnotic trance who painlessly are stabbing knives or lances into their body in expectation of divine assistance, grace, or forgiveness from sins. Further impressive examples are experiments where volunteers showed poor perception and identification of figures presented on a screen in front of them when A) they were hypnotically suggested that their view of the screen would be blocked by a virtual wooden board [10] or B), where color pictures were hypnotically suggested being seen in black and white. Here, subjects visual processing areas for black and white information became activated instead of areas for color vision [11].

Since the induction of auditory deafness with hypnotic procedures is considered to be rather difficult (see f.e. [12]), only a few studies were published so far. Unfortunately, their results are anything but clear [13–18]. While some claimed positive effects, others argued rather doubtfully that the result more likely mirror deception, simulation, or suggestion-induced reporting biases. Erickson's studies [13, 14] offer a number of explanations why suggestions of deafness work. He pointed to habituation, receptor adaptation, inattentiveness or deep trance, to mention a few, but beyond the description of participants' statements none of these explanations was rigorously tested in the two investigations. Dynes [15] explicitly excluded any physiological explanation and suggested that hypnotically induced deafness might reflect a consequence of cortical deactivation associated with reduced consciousness or dissociation within the central nervous system. Barber et al. [16] did not observe significant differences in reported deafness or loudness of tones by participants who either received hypnotic deafness suggestions or only were told to experience deafness without being suggested deafness while hypnotized. Due to similar effects in non-hypnotized participants, they concluded that it is not necessary to administer a procedure traditionally subsumed under the term 'hypnotic induction' to elicit deafness-like responses. Positive and negative auditory hallucinations can be elicited by administering brief task-motivating instructions by telling subjects that they can perform well and best of their abilities on suggested tasks. I.e., suggested deafness was interpreted as the result of suggestion-induced reporting biases and thus as a consequence of primarily socio-cultural expectations, attitudes, and beliefs [19–22]. The belief in the suggested experiences and the expectations raised by suggestions are considered to primarily determine the respectively reported concrete hypnotic experience. Hypnosis was thus interpreted as similar in its function to placebos or nocebos [22] and originated by expectations and believes in the occurrence of a predicted experience. Spanos et al. [18] interpreted their observations in a similar manner and concluded that their high susceptible subjects seemed having believed more in the experience of deafness at the end of the experiment than not to have heard anything during each trial of the experiment and for this reason indicated that they had heard nothing or less. High susceptibles were characterized as particularly vulnerable that such expectations are more likely to apply than what one has actually experienced. These observations were replicated and supported by two other studies [23, 24] and again, it was stated that research provided strong support for the hypothesis that reports of suggestion-induced perceptual alteration proffered by highly hypnotizable hypnotic subjects reflect, to a substantial degree, reporting biases rather than actual changes in perceptual processing. Barber and coworkers [25–27] as well as Spanos and colleagues [19, 24] furthermore pointed out that such reporting biases could become amplified if the test situation was explicitly named „hypnosis”or participants were explicitly told that they will be put into hypnotic trance before they were exposed to hypnotic or non-hypnotic suggestions and told that the result of their activities during the test would strongly depend on their engagement, motivation, compliance, and role-taking [28].

The present investigation builds on these previous studies on hypnotic deafness and extends this research by asking how the brain reacts to hypnotic deafness suggestions as made visible by auditory event-related potentials (ERPs) and activities of their underlying cortical sources derived from the electroencephalogram. According to carefully searches with PubMed and Web of Science, only one study [29] so far used ERP measures that assessed ERPs related to information processing of tone pips, i.e., the so called P300 component of late auditory ERPs. Tone pips were presented either when subjects were A) exposed to hypnotic suggestions that hearing the tone pips would be obstructed due to virtual foam earplugs in their ears, B) that they should let themselves become completely deaf and C) exposed to a waking control condition where no hypnotic trance was induced and no suggestions regarding the tones were given. Results indicated: A) smaller P300 amplitudes in high susceptibles while experiencing the obstruction suggestion as compared to their own waking condition and to the P300s of low susceptibles and B) larger P300-amplitudes while experiencing the deafness suggestion again as compared to the P300 amplitudes of their own waking condition and to P300 of low susceptibles.

The present study used an auditory three-stimulus oddball paradigm [30] and EEG multichannel recordings (96 channels) to analyze the magnitudes of the N1 component and the magnitudes and topographies of the P3b component and its neural sources. Furthermore, we also investigated several behavioral responses of high and low susceptible participants to the three different auditory stimuli.

The N100 or N1 component is a large, negative-going late ERP component that commonly peaks between 80 and 120 milliseconds after stimulus-onset (for references for this and the following statements see [31–33]). It is distributed commonly over frontocentral brain areas. Induced by any task-relevant and any unpredictable task-nonrelated stimulus, the auditory N1 is generated by a network of neurons in the primary and secondary/associative auditory cortices of the superior temporal gyrus, Heschl's gyrus, and in the planum temporale. It also could be generated in frontal and motor areas. Generating structures are larger in the right than in the left hemisphere. The N1 is preattentive and involved in the allocation of selective attention for tone frequency and tone-pattern discrimination and also for the identification of loudness and the timing of tones. Earlier studies by our group in response to painful and non-painful somatosensory stimuli or visual stimuli revealed that the N1 reflects early attentional responses towards physical aspects of stimuli and processes for stimulus discrimination [34–39]. Furthermore, we also showed that these processes were not affected by hypnosis [10, 40–43]. If the same holds true for the auditory modality, we expected the same magnitude and source configuration of the N1 component under hypnotic deafness suggestion as in the control condition described below.

The positive-going P3b represents a prominent brain-wide activity that was heavily investigated so far and related to cognitive processes associated with the analysis of stimulus probability and the recognition and categorization of stimuli [44, 45]. Verleger [46] has recently also strongly emphasized, that the P3b amplitude is an important signature for the association of a stimulus to task relevancy. All of these cognitive processes include attentional processes and comparison to and updating of internal representations of stimuli, i.e., long-term and especially working memory functions. According to the triarchic concept of the P3b, suggested by Johnson [47], the magnitude of P3b amplitude further was shown to vary as a function of stimulus distinctiveness and subject’s attention (E), stimulus probability P, and meaning or task relevancy (M) of a stimulus according to the following formula: [P3bamp = f(E x (P + M))]. In a previous study, we observed P3b reductions after a visual blocking suggestion [10]. In the present transfer of this study to the auditory modality we expect reduced P3 amplitudes and changes in source activities under hypnotic deafness suggestion compared to the control condition described below.

In the present within-subjects designed study, each participant was exposed to four different experimental conditions. During condition A (real hypnosis, HYP), she/he first received suggestions to enter hypnotic trance and then was exposed to a series of auditory deafness suggestions while hypnotized and stimulated with three auditory Oddball stimuli. In condition B (hypnosis simulation, SIM), she/he received no trance induction, but was requested to simulate being hypnotized and not to hear the auditory Oddball stimuli. In condition C (disattention, DIS), no trance induction was offered but each participant was instructed to focus on a film clip and to ignore all auditory stimuli (same stimuli as in A). Finally, in condition D (control, CON), each participant received the same stimuli as in A, but without any starting trance induction or any deafness suggestion. Thus, the present study tested:

[1] as to what extent the perception of acoustic stimuli as indicated by loudness, hit rate, event-related activity, ERP-topography, and its underlying neural sources can be significantly affected by hypnotic deafness suggestions and [2] whether theses effects differ from the SIM and DIS condition. [3] Are these intervention effects modulated by susceptibility? [4] Does the analysis of neural activities uncover cognitive mechanisms that cannot be derived simply from participants’ behavioral responses?

## Materials and methods

### Subjects and data

Forty-eight healthy volunteers (24 females, mean age 24.5 years, age range 19–54 years; 24 males, mean age 26.3 years; range 18–46 years) participated in the study. Participants were recruited at the Friedrich Schiller University and assigned to two equally-sized subgroups of 24 individuals each (12 females) according to their susceptibility score as examined prior to the experiment by the German version of the Harvard Group Scale of Hypnotic Susceptibility (HGSHS; [48]). Participants with scores between zero and three were assigned to the low susceptible group and those with scores between 8 and 12 to the high susceptible group. Participants either received course credits for participation or a financial bonus of 42€ (€ 10/hour). The study protocol was approved by the ethics review board of the Faculty of Social and Behavioral Sciences of the University of Jena and was in line with the declaration of Helsinki.

### Procedure and stimuli

The four experimental conditions were applied in one experimental session. To minimize sequence-/crossover-effects of experimental conditions, their order was counterbalanced across participants. At the beginning of the experiment, participants received detailed information about the research questions and the different sections of the experiment. They were told that the main research question of the study was to investigate how different parts of the human brain get affected by different tones and how hypnosis (HYP), simulation of hypnosis (SIM), and disattention (DIS) would modify participants’ behavioral and brain responses compared to a control condition (CON). Afterwards, the conditions and stimulus application procedure were explained and open questions answered in detail. Participants then signed the informed consent. To provide an experience to which participants could refer to the deafness suggestions, they put on earplugs and tested their effect on hearing. While exposed to the experimental conditions, participants were seated on a comfortable chair in a shielded and dimly lit EEG chamber. An EEG-electrode cap with 96-sintered Ag/AgCl electrodes (EASYCAP GmBH, Herrsching-Breitbrunn, Germany) that were topographically placed about the same distance apart for each other was fixed on participants’ scalp. An additional electrode was fixed under the lower lid of the left eye to record the vertical electrooculogram (EOG). Then participants were familiarized with the behavioral response button at the armrest of the chair and with the tones preseted during the experiment.

In each of the experimental conditions, a three-stimulus-oddball paradigm was applied [49]. This paradigm presented a random sequence of 500 tones of three different frequencies and stimulus probabilities, hereafter termed as standard, distractor, and target stimuli. Frequencies of standards, distractors, and targets were 500, 400, and 600 Hz, presented with probabilities of 0.8, 0.1, and 0.1, respectively. Each stimulus was applied via current-free in-earphones (E-A-RLINK™, 3M Company, Indianapolis, USA) for 100 ms with rise/fall times of 10 ms and an average inter-stimulus interval of 1.5 secs. To secure that participants perceived the tones equally loud, individual sound thresholds were examined prior to the experiment. In all experimental conditions, participants were requested to press the response button whenever a target was perceived and to ignore distractor and standard tones. These button presses served to determine the target hit-rate and the latency of responses in ms to each target stimulus. Furthermore, following each condition, participants were asked how loud they perceived the three different tones using a Likert Sale rating from 0 (not heard) to 20 (very loudly heard). The duration of the oddball task was 15 minutes in each condition.

The HYP condition was induced by application of item 1 of the Stanford Hypnotic Susceptibility Scale, form C ([50]; for wordings see S2 File) and additional instructions as presented in S2 File. Then suggestions followed that an earplug of a little cotton ball put into both outer-ear canals would obstruct the perception of any tone (see S2 File) but still allow to follow the voice and instructions of the hypnotist. The suggestion was repeated after every 100th tone.

In the SIM condition, participants were requested to pretend being hypnotized and not hearing any tones. To assist the role-play of simulation and participants’ compliance with the instructions and tasks, they were requested to remember how they behaved during the preceding HGSHS-test session some days before the experiment proper or during any earlier experience with hypnosis (e.g., to the hypnosis experience of the present experiment (HYP) that might have preceded the SIM condition; for wording of this condition see the S2 File). Additionally, they were also told being recorded by a camera, and later rated for the quality of their role-play by an expert of hypnosis. The three most successful pretenders were promised a bonus between 10 to 30€. During Oddball presentation, participants were repeatedly reminded to simulate being hypnotized and not hearing any tones.

During the DIS condition, participants where requested to attentively watch the soundless movie “The way things go” by Fischli and Weiss [51] during stimulation with the tones and not to focus on the tones but instead focus on the film and memorize as many of its details as possible. They were informed that each correct answer would be rewarded with 50 cents during a test session at the end of this experimental condition. In the CON condition, participants were asked to sit calmly in the chair while stimulated with the tones.

For testing of the quality of trance induction during HYP, item one of the SHSS [50] was applied. Lowering of the arm at least for 10 cm was scored with ‘1’ and with ‘0’, if lowered less than 10 cm (Hypnosis Test). Additionally, participants were asked, how well they managed not to hear the stimuli during the deafness suggestions at the end of the HYP condition (see S2 File; Hypnosis Score). At this time point, participants also received the German version of the Inventory Scale of Hypnotic Depth (ISHD; [52, 53]) that assessed participants’ depth of hypnotic trance.

At the end of the SIM condition, participants were asked how much they felt hypnotized by using a dichotomous scale with score ‘1’ indicating ‘well hypnotized’ and score ‘0’ ‘not at all hypnotized’ (SIM Hypnotized). Furthermore, quality of simulation was assessed offline by a blinded observer using 4 rating scales that provided a maximal behavior-score of 8 different behaviors displayed by participants during HYP (Simulation Score, details see S2 File).

At the end of the DIS condition, participants were asked about details of the film using a 10-item memory questionnaire (Distraction Score, for details see S2 File) and promised a reward of 50 cents for each correctly answered content question ([51]; for wording of the instruction of this condition also see the S2 File). Furthermore, they were requested to rate how much they felt distracted by the film, again using a 10-point Likert-Scale (0–10, Movie Distraction).

Finally, in addition to the loudness of each tone, participants were also requested to rate their valence and arousal using the SAM procedure [54]. These scales will not be reported in this paper.

Furthermore, at the beginning of each experimental conditions, spontaneous task-unrelated Rest-EEG activities were recorded with eyes open for a period of 2 min. These recordings were also repeated at the end of each condition and a second Rest-EEG recording was obtained at the end of the hypnosis induction before the start of the hypnotic suggestions in condition HYP. Finally, a second experimental section followed each oddball task in a second part of each condition, where auditory steady-state stimuli were presented. However, the two latter experimental sections are not subject of this paper. The experiment was programmed and presented by means of Presentation® software (Version 17.1, Neurobehavioral Systems, Inc., Berkeley, CA, www.neurobs.com).

Additionally, a high‐resolution T1‐weighted anatomical volume (208 slices, TR = 2400 ms, TE = 5 ms, flip-angle = 8°, matrix = 300 × 320 mm, resolution = 0.8 × 0.8 × 0.8 mm) was acquired for each participant using a 3-Tesla magnetic resonance scanner (Tim Trio, Siemens, Medical Systems, Erlangen, Germany) and employed for source reconstruction of EEG data.

### EEG recording and preprocessing

During EEG recording, all channels were referenced online to the nose tip. Impedances of all electrodes were kept below 10 kΩ. EEG signals were registered using BrainAmp amplifiers and the BrainVision Recorder software (both Brain Products, Gilching, Germany). Following analogue band-pass filtering (0.015–250 Hz), continuous EEG signals were digitized with a sampling rate of 1 kHz and stored to hard disk for later offline analysis. EEG data were preprocessed using EEGLAB ([55], Version 13.6.5b). For further processing, datasets were down-sampled to 250 Hz and re-referenced to linked mastoids. Datasets were pruned from artifacts related to eye-blinks and ocular movements using independent component analysis (ICA). Therefore, a duplicate of the re-referenced EEG dataset was offline band-pass filtered (*pop_eegfiltnew*) from 1−40 Hz with a transition bandwidth of 1 Hz (highpass) and 10 Hz (lowpass), respectively, using a Hamming windowed sinc finite impulse response (FIR) band-pass filter and subsequently segmented into continuous 1 s intervals. This dataset was then pruned from unique, nonstereotyped artifacts by applying a higher order statistic function (*pop_jointprob*) to each electrode channel to discard data segments containing unlikely EEG values (> ±3SD) that are indicative of artifacts [56]. Extended infomax ICA was then applied to the pruned dataset.

The original re-referenced EEG dataset was filtered offline using a Hamming windowed sinc FIR band-pass filter with a transition bandwidth of 0.1 Hz (highpass) and 10 Hz (lowpass), respectively. Subsequently, the ICA demixing matrix was applied to this dataset. Components representing eye-blinks or ocular movements were identified using the *EyeCatch* precedure [57] and subtracted from data [58]. The dataset was segmented into epochs from −0.2 to 1.0 s relative to stimulus onset and baseline corrected using the average activity of each single EEG channel/participant/condtion of the pre-stimulus interval from −0.2 to 0 s. The epochs were pruned from non-stereotyped artifacts (*pop_jointprob*) by discarding epochs with amplitude values greater than ±3 SD. On average, 83.5% (Min: 68.9%, Max: 90.7%) of all trials were retained after artifact rejection. In the four experimental conditions (HYP/CON/DIS/SIM), the mean number of trials amounted to ≈41/≈40/≈41/≈43 valid target trials (*Min*: 25/26/28/27, *Max*: 50/48/50/50), ≈42/≈41/≈42/≈44 distractor trials (*Min*: 32/28/29/33, *Max*: 50/49/49/49), and ≈333/≈322/≈332/≈351 standard trials (*Min*: 234/205/252/253, *Max*: 386/393/392/391) for each subject. ERP waveforms were averaged separately for each participant, stimulus-type and experimental condition. These preprocessed datasets were used for the standard ERP-analyses (single electrode analysis) and imported into SPM12 (v7219; http://www.fil.ion.ucl.ac.uk/spm) for EEG topography-by-time-cluster-analysis and source analysis [59].

### Distributed source reconstruction

For EEG source reconstruction, we employed the parametric empirical Bayesian (PEB) framework and the multiple sparse priors (MSP) method as implemented in SPM12. The individual T1-weighted anatomical volume was used for computing the forward model of each participants’s brain. The head model comprised four meshes based on the cortex, inner skull, outer skull, and scalp. The distributed model approach constrained the source space to 8196 vertices (4098 per hemisphere) of the cortical surface mesh. The EEG spatial sensor locations were recorded with Polhemus™ Fastrak (Colchester, Vermont, USA, www.polhemus.com) system and mapped to the coordinate system of each participants’ anatomical MRI image. A lead field matrix (forward solution) then was computed using a three-shell Boundary Element Model (inner skull, outer skull, and scalp meshes). Prior to source estimation, spatial and temporal data reductions were conducted to increase the signal-to-noise ratio (SNR). Subsequently, datasets were subjected to group-based source reconstruction using the MSP-approach as inversion type and Greedy Search as the fitting algorithm. The time window of inversion ranged from −100 to 600 ms. The results of source estimation were averaged over the P3b window (320−470 ms), and exported to MNI brain space as surface-based GIFTI images. For each subject, a set of 4-by-3 (condition, Stimulus-Type) GIFTI images based on the P3b window was then created and used for statistical analysis (see below). For further details on source reconstruction see the S1 File.

### Statistics

#### Behavioral data

To analyze how well the participants managed to distinguish the ‘signal’ (target stimuli) from ‘noise’ (standards and distractor stimuli), we calculated the discriminability index (*d-*prime) for each participant in each experimental condition as well as the receiver operating characteristic (ROC) curves, and the area under the ROC curve (AUC). A two-way analysis of variance (ANOVA) was conducted to test the effects of Condition (CON, DIS, HYP, SIM) and Susceptibility (low vs. high) on *d-*prime values. Additionally, reaction times to targets were statistically analyzed using linear mixed-effects modeling (LMEM). To this end, we employed the *lmer* function of the *lme4* package [60] for estimating fixed and random coefficients. The model was fitted by restricted maximum likelihood estimation (REML) using Condition, Susceptibility, and the two-way interaction as fixed effects and subject as random effect. Furthermore, a three-way ANOVA was carried out on loudness ratings by Condition, Susceptibility, and Stimulus-Type (standard, distractor, target).

For each analysis, the significant interaction of Condition-by-Susceptiblity was followed by 5 defined post-hoc tests (CON vs. DIS, CON vs. HYP, CON vs. SIM, DIS vs. HYP, and HYP vs. SIM) applied to low and high susceptibles separately. Post-hoc tests, i.e., a family of 10 tests for each analysis, were Bonferroni-adjusted to counteract the problem of multiple comparisons. We also conducted between-group differences in each condition using independent *t*-tests. We considered *p*-values < .05 to be statistically significant. All statistical analyses were carried out in R (version 3.6.2, [61]).

#### Sensor-level analysis

The sensor-level analysis was conducted separately for the N1 (80−168 ms), and P3b (320−470 ms) windows using two different approaches. Approach number one, the *Single-Electrode-Analysis*, used the common, traditional concept of ERP analysis and focused on two individual electrodes used as standards in countless former ERP studies, i.e. the frontocentral electrode Fz for the N1 and the centroparietal electrode P29 that comes closest to the electrode Pz, considered the standard electrode for the investigation of most cognitive functions and processes associated with the P3b component. For this electrode, we examined the effects of hypnotic suggestions, distraction, and simulation in relation to the control condition and the effects of stimulus probability of the three stimuli as central brain electrical parameters according to the triarchic model of P300 amplitude (for more details see below). Each participant’s event-related averaged voltage-time wave at this electrode E29 that was preprocessed according to the procedure outline above then was used as basis for all succeeding N1 and P3b amplitude analyses using a three-way ANOVA (Condition, Stimulus-Type, and Susceptibility). Significant differences between theses factors were considered as true, when their *p*-values were smaller than .05 and the statistics survived the Bonferonni post-hoc test and/or the Greenhouse-Geisser corrections of degrees of freedom due to lack of data sphericity in repeated measures ANOVAs, where applicable. Additionally, we calculated cortical voltage maps across all 96 EEG-electrodes for visual inspection of topographical differences of averaged N1 and P3b amplitudes between the two groups, the four experimental conditions, and the three Stimulus-Types.

The second sensor-level ERP data analysis was based on the *Topography-by-Time-Cluster-Analysis* (TTCA) as implement in SPM12. This analysis of ERP activities extended the single electrode approach of the first analysis to an analysis of topographical activity of all 96 electrodes at all single sample points within the above outlined windows of N1 and P3b amplitudes. While the classic analyses of N1 and P3b activities based on a single electrode only provides insights into a very small, selective neuronal process of the brain, the TTCA considers extended neural activity clusters and their topographical dynamics within the observation time windows. Therefore, for each subject, a set of 4-by-3 (Condition, Stimulus-Type) NIFTI images (2D sensor-space x time volumes) was created for the respective latency window and employed for statistical analyses.

#### Statistical analysis of sensor- and source-level data

For group statistical analysis of the 2-by-4-by-3 design (Susceptibility, Condion, Stimulus-Type), we opted for the partitioned error approach (random effects analysis). The NIFTI (sensor-level)/GIFTI (source-level) images were first transformed into a set of differential effects for each subject, i.e., 1st level contrast images (within-subject analysis) were created using the *ImCalc* facility in SPM12. This resulted in four sets of 1st level contrast images to test for three main effects, three 2-way interaction effects and one 3-way interaction effect. For each set of contrast images, two General Linear Models (GLM) were specified (using one-/two-sample *t*-test designs or 1-way ANOVA designs) in SPM12, and estimated at the 2nd level, and relevant *t*- or *F*-contrasts were specified to test for the effects of interest. The significant interaction effect was followed by focused contrasts (simple effects) by specifying a 4-by-3 (Condition, Stimulus-Type) repeated measures ANOVA within the GLM framework of SPM12. To improve sensitivity of contrasts, we added 48 subject columns into the GLM that serve to remove between-subject variance. For further details on GLM specification and testing see S1-1 & S1-2 Tables of S1 File. Effect sizes for within-subject ANOVA designs, expressed as partial eta squared ${(\eta }_{p}^{2})$, were calculated from the *t*-contrast (*t*-value) and its degrees of freedom using the formula: $\eta_{p}^{2}=\frac{F*{df}_{effect}}{F*{df}_{effect}+{df}_{error}}$ [62], where *F*(1, df) = *t*-value^2^.

Since repeated measure group designs might be confounded by possible order and sequence effects, attrition, and fatigue, Shadish, Cook [63] suggest to (a) counterbalance the experimental conditions across subjects and/or (b) incorporating the order effect into the design and controlling it statistically. The application of this suggestions revealed no statistical evidence of order and sequence effects for behavioral and EEG sensor parameters of interest of the present study.

## Results

### Extent of hypnotization, simulation, and distraction

High susceptible subjects differed significantly from low sugestibles in the Hypnosis Test applied during the hypnosis induction (χ^2^_1_ = 9.6, *p =* .*002*). Based on the odds ratio, the odds of high susceptibles to lower the hand (Hypnosis Test) were 16.6 times higher than in low susceptibles. The Hypnosis Score, indicating how well deafness was realized while being hypnotized during the HYP condition, was significantly larger in high than in low susceptibles (*M* = 2.9 vs. 2.1, *t*_46_ = 3.0, *p =* .*005*). Results of the ISHD further revealed significant differences of the experienced depth of trance between low and high susceptible participants (F_1,46_ = 24.7, *p* = < .001, η^2^ = 0.35). Three particpants of the high susceptible group reported deep trance, 19 participants a medium level of trance, and two participants not having got into trance during HYP. In contrast, among low susceptibles, none experienced deep trance, 8 a medium level of trance and 16 did not get into trance at all during HYP. Item 8 of this questionnaire further indicated that high susceptible participants perceived the hypnotist’s voice much farer away (*M* = 2.5, *SD* = 0.1) than low susceptibles (*M* = 1.9; *SD* = 0.9, *t*_1,46_ = 2.57; Cohen’s *d* = 0.74). ISHD total score also correlated nicely with participants’ HGSHS scores (Pearson’s *r* = 0.7, *p* = < .001).

No significant differences between high and low susceptible subjects were observed for the Simulation Score (highs/lows: *M* = 5.7 vs. 5.2), i.e., the external rating of participants’ simulation achievement, for the SIM Hypnotized, i.e., whether participants felt being hypnotized (highs/lows: 54% vs. 42%) or not during the SIM condition, for the Distraction Score, describing how many details about the film were remembered (highs/lows: *M* = 7.0 vs. 7.4), and for the Movie Distraction score, indicating how the participants felt distracted by the film retrospectively in the DIS condition (highs/lows: *M* = 6.2 vs. 5.5).

### Behavioral data

#### Target detection accuracy

**Fig 1:**
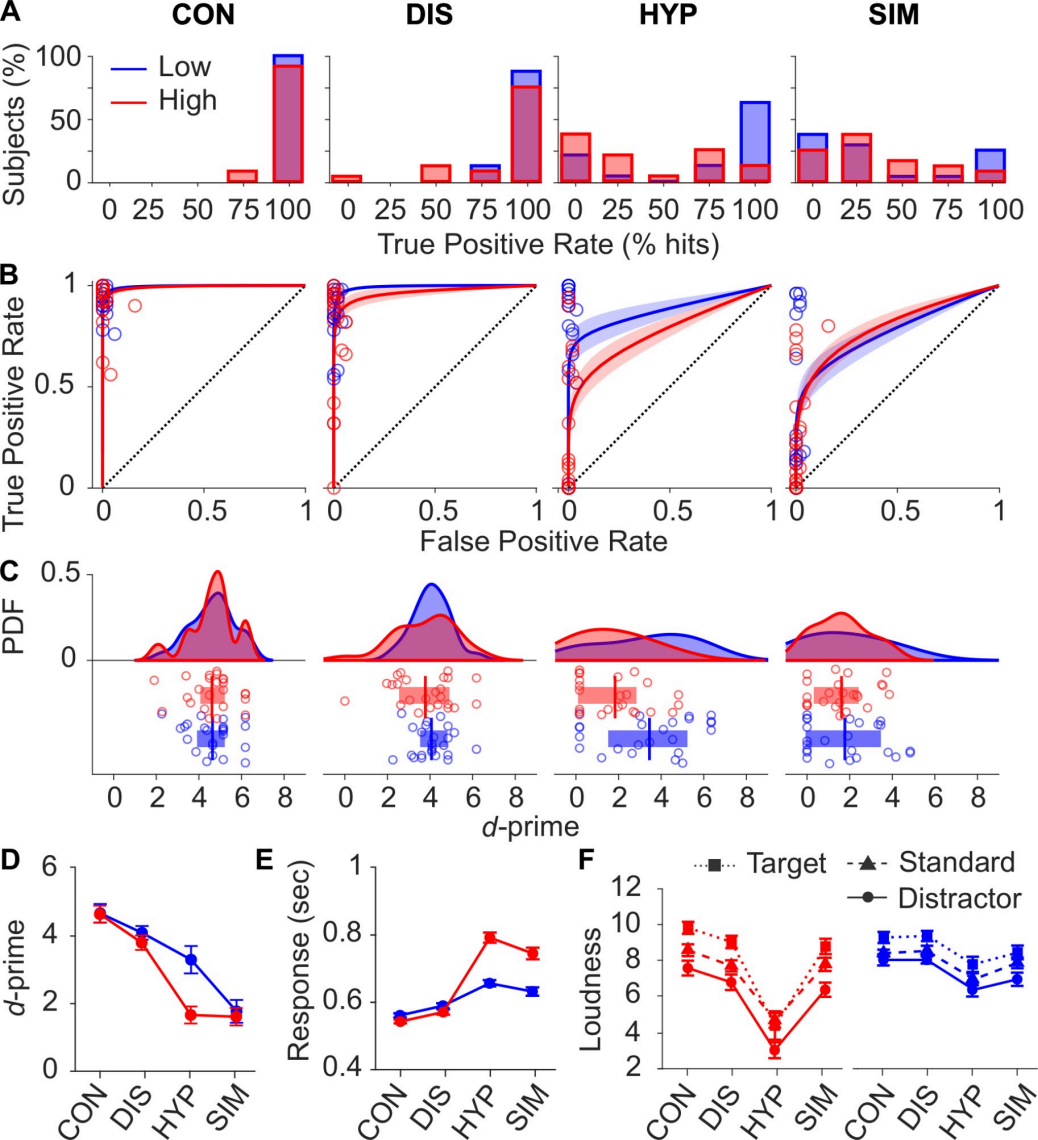
**Behavioral data are depicted separately for low (blue), and highly (red) susceptible participants in control (CON), distraction (DIS), hypnosis (HYP), and simulation (SIM). (A)** Histograms of hit rates showing the percentage of subjects in each of the five mutually exclusive hit rate classes (0≤25≤50≤75≤100%). **(B)** Grandaverage receiver operating characteristic (ROC) curves in the four conditions. Circles mark the empirical true positive (hit) rates plotted against false positive (false alarm) rates of each subject. Shaded areas indicate the 95% CI. A curve above the dotted diagnol represents a discrimination better than random. **(C)** Probability density function (PDF) and scatter plot of estimated *d*-primes. Vertical lines and boxes mark the mean and interquartile range (difference between 75^th^ and 25^th^ percentiles) of estimated *d*-primes, respectively. **(D)** Line plot of *d*-prime (mean ± within-subject standard error). **(E)** Target response time (mean ± within-subject standard error). **(F)** Loudness rating of target, standard, and distractor stimuli (mean ± within-subject standard error).

Fig 1A–1C depicts the true positive rates (hits), the receiver operating characteristic (ROC) curves, the probability density function, and scatter plots of *d*-primes for each condition, separately for low and high susceptibles. On average, participants detected 92% of the target stimuli (true positive rate /hits) in control (CON), 83% in distraction (DIS), 48% in HYP, and 29% in SIM (Fig 1A) with a false positive rate (false alarms) of 1% in each condition. With regard to the ROC curves, low and high susceptible participants showed nearly ideal target detection accuracy in CON as indexed by the area under the ROC curve (AUC = 0.99), followed by DIS (AUC = 0.98), HYP (AUC = 0.83) and SIM (AUC = 0.79) conditions (Fig 1B). The estimated *d-*prime scores were subjected to a two-way ANOVA with the within-subjects factor Condition and the between-subject factor Susceptibility (low, high). Accuracy in target detection varied significantly across the levels of Condition (*F*_2.5, 114.5_ = 46.4, *p* < .001, $\eta_{p}^{2}$ = 0.50). Additionally, we observed a significant interaction of Condition by Susceptibility (*F*_2.5, 114.5_ = 3.5, *p* = .02, $\eta_{p}^{2}$ = 0.07) indicating that *d*-prime differences between the conditions varied between low and highly susceptibles (see Fig 1C and 1D). Post-hoc tests for highly susceptibles revealed significantly larger *d-*primes in CON vs. HYP (*t*_184_ = 7.0, *p = <* .001), CON vs. SIM (*t*_184_ = 7.1, *p = <* .001), and DIS vs. HYP (*t*_184_ = 5.0, *p = <* .001), whereas no significant differences were observed for CON vs. DIS (*t*_184_ = 2.0, *p =* .25), and HYP vs. SIM (*t*_184_ = 0.1, *p =* .91). For low susceptibles, *d-*primes were significantly larger in CON vs. HYP (*t*_184_ = 3.2, *p =* .008), CON vs. SIM (*t*_184_ = 6.8, *p = <* .001), and moreover in HYP vs. SIM (*t*_184_ = 3.6, *p =* .002), whereas no significant differences were observed for CON vs. DIS (*t*_184_ = 1.4, *p =* .89) and DIS vs. HYP (*t*_184_ = 1.9, *p =* .32). Furthermore, accuracy of target detection was generally lower for high susceptibles compared to low susceptibles as indicated by the main effect of Susceptibility (*F*_1, 46_ = 4.4, *p* = .04, $\eta_{p}^{2}$ = 0.09; see Fig 1D). Between-group comparisons (high vs. low) of *d*-primes revealed only a significant difference for HYP (*t*_46_ = –3.2, *p =* .001).

#### Reaction time to target

Additionally, we investigated whether reaction times to target stimuli differed between conditions and low and high susceptibles. To this end, a linear mixed-effects model was fitted (LMEM) to the data using Condition, Susceptibility, and the two-way interaction as fixed effects and subject as random effect. Fig 1E shows the mean response times to the target stimuli for low and high susceptibles and each condition. The LMEM analysis of response times revealed a significant fixed-effect for Condition (F_3, 28.3_ = 22.5, *p* < .001) and a significant interaction of Condition by Susceptibility (F_3, 28.3_ = 3.7, *p* < .023). Post-hoc tests revealed that highly susceptibles responded significantly faster to target stimuli in CON vs. HYP (*M =* −251 ms, *t*_5984_ = −7.2, *p = <* .001), CON vs. SIM (*M =* −225 ms, *t*_5984_ = −6.2, *p = <* .001), and DIS vs. HYP (*M =* −218 ms, *t*_5984_ = −5.4, *p = <* .001), while there was no significant difference for CON vs DIS (*M =* −33 ms, *t*_5984_ = −1.8, *p =* .69), and HYP vs SIM (*M =* 26 ms, *t*_5984_ = 0.78, *p =* .99). Low susceptibles responded significantly faster in CON vs. HYP (*M =* −100 ms, *t*_5984_ = −3.3, *p =* .009), while there was no significant difference for CON vs DIS (*M =* −32 ms, *t*_5984_ = −1.8, *p =* .72), CON vs. SIM (*M =* −99 ms, *t*_5984_ = −3.3, *p =* .06), DIS vs. HYP (*M =* −68 ms, *t*_5984_ = −1.9, *p =* .54), and HYP vs SIM (*M =* 1 ms, *t*_5984_ = 0.03, *p =* .99). Between-group comparisons (high vs. low) of response time revealed significant differences for HYP (*M* = 121 ms, *t*_32_ = –2.1, *p =* .04) and SIM (*M* = 161 ms, *t*_31_ = –2.7, *p =* .01). It is to note that degrees of freedom vary compared to total sample size but this is due to the fact that some participants did not respond to any target.

#### Loudness of stimuli

Loudness ratings were subjected to a three-factor mixed-design ANOVA with the Condition, Stimulus-Type, and Susceptibility. The analysis revealed a significant main effect of Condition (*F*_2.4, 111.8_ = 31.7, *p* < .001, η^2^ = 0.11) and significant interaction effect of Condition by Susceptibility (*F*_2.4, 111.8_ = 8.4, *p* < .001, η^2^ = 0.03) indicating that differences between conditions for low and highly susceptibles varied, see Fig 1F. Highly susceptibles rated loudness of stimuli in HYP significantly lower compared to CON (*t*_138_ = −9.5, *p = <* .001), compared to DIS (*t*_138_ = −7.8, *p = <* .001) and compared to SIM (*t*_138_ = −7.4, *p = <* .001), while there was no significant difference between CON vs. DIS (*t*_138_ = 1.7, *p =* .98), and CON vs. SIM (*t*_138_ = 2.0, *p =* .41). Low susceptibles rated loudness of stimuli in HYP significantly lower compared to CON (*t*_138_ = −3.1, *p =* .04), and compared to DIS (*t*_138_ = −3.2, *p = <* .03), while there was no significant difference between CON vs. DIS (*t*_138_ = −0.1, *p =* .99), CON vs. SIM (*t*_138_ = 1.7, *p =* .98), and HYP vs. SIM (*t*_138_ = −1.4, *p =* .99). Also, there was a significant main effect of Stimulus-Type (*F*_1.5, 68.8_ = 40.5, *p* < .001, η^2^ = 0.04) indicating that loudness ratings varied between stimuli; targets were rated not significantly louder than standards (*M =* 8.4 *vs*. 7.6, *t*573 = 2.2, *p =* .06*)*, but the standards compared to distractor stimuli (*M =* 6.6, *t*573 = 2.5, *p =* .03*)*. Between-group comparisons (high vs. low) of loudness revealed only significant differences for each stimulus in HYP (target: *t*_46_ = –2.7, *p =* .01; distractor: *t*_46_ = –2.3, *p =* .02; standard: *t*_46_ = –3.7, *p =* .001).

### Sensor analysis

**Fig 2:**
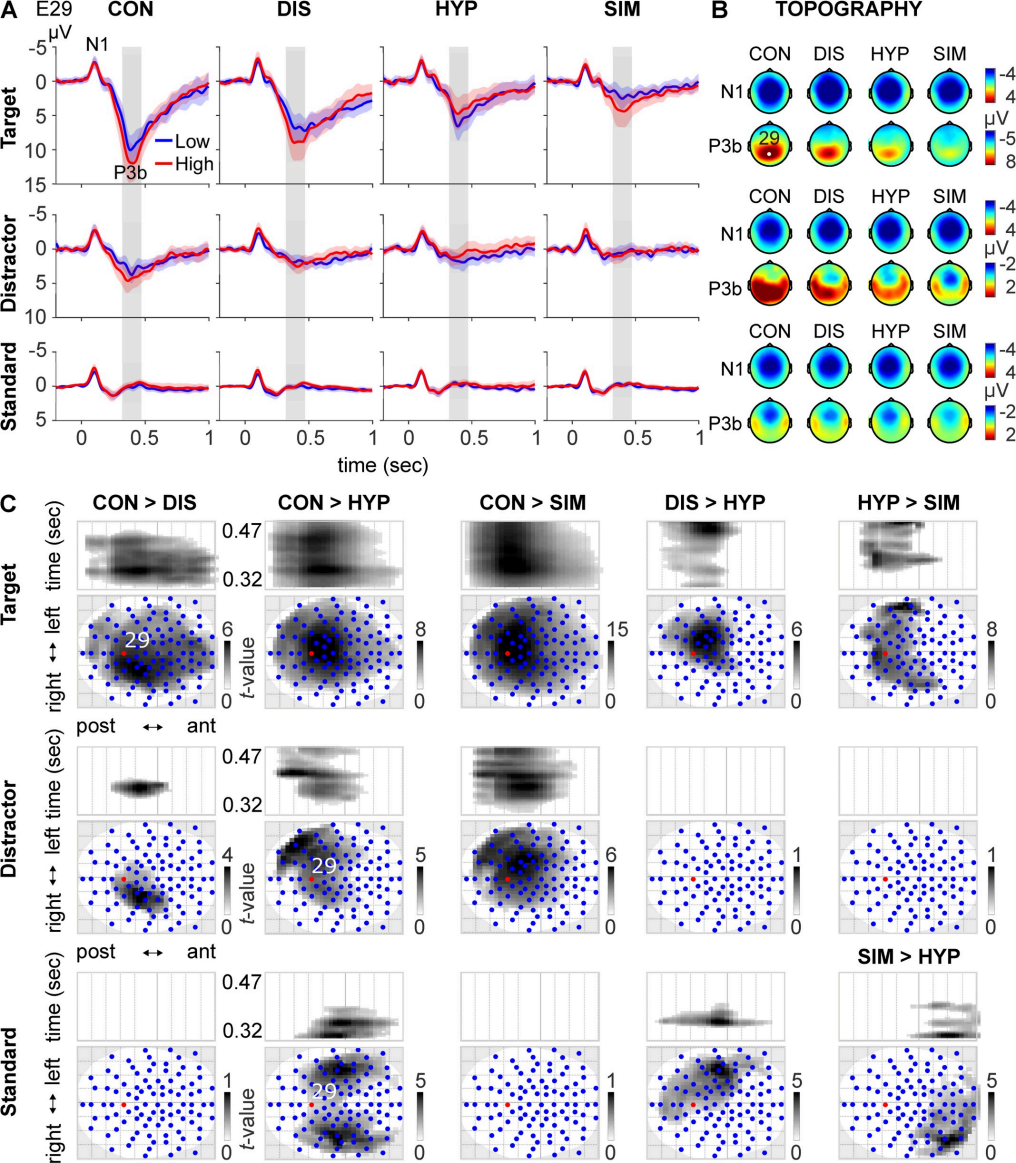
**(A)** Grandaverage waveforms and 95% C.I. in control (CON), distraction (DIS), hypnosis (HYP), and simulation (SIM) for target, distractor, and standard at the posteriocentral electrode 29 for low (blue, *n* = 24) and highly susceptible (red, *n* = 24) participants. The grey rectangle marks the P3b time window (320–470 ms) used for statistical analysis of P3b amplitudes at sensor-level. **(B)** Topographical maps of scalp voltage at the peak latency of the target N1 (100 ms) and P3b (400 ms) depicted for condition and stimulus-type across all subjects. **(C)** Focused contrasts within the Topography-by-Time-Cluster-Analysis to disentangle the interaction of condition by stimulus-type. There were no significant amplitude differences for the distractor between DIS and HYP as well as HYP and SIM, and for the Standard between CON and DIS and between CON and SIM. The summary statistic scalp-time images were thresholded at *p* < .001 (uncorrected) with FWE correction at cluster-level, *p* < .0017 (two-tailed, Bonferroni-adjusted, *n =* 15), based on random field theory. The statistical parametric maps (SPMs) are displayed as Maximum Intensity Projection (MIP) of the 3D (scalp x time) summary statistic image. Blue dots mark the electrode sites. post = posterior; ant = anterior.

Next, we analyzed whether the different experimental conditions affected participants concomitant neural processes differently in terms of the N1 and P3b component. Fig 2A displays the grandaverage waveforms at the posteriocentral electrode 29, approximately matching the Pz electrode of the International 10–20 system, for the target, distractor, and standard in CON, DIS, HYP, and SIM for low (blue) and highly susceptibles (red). The scalp voltage topograhies at the peak latency of the N1 (100 ms) and P3b (400 ms) are depicted in Fig 2B for each condition and stimulus-type across all subjects.

#### Single electrode analyses

**Fig 3:**
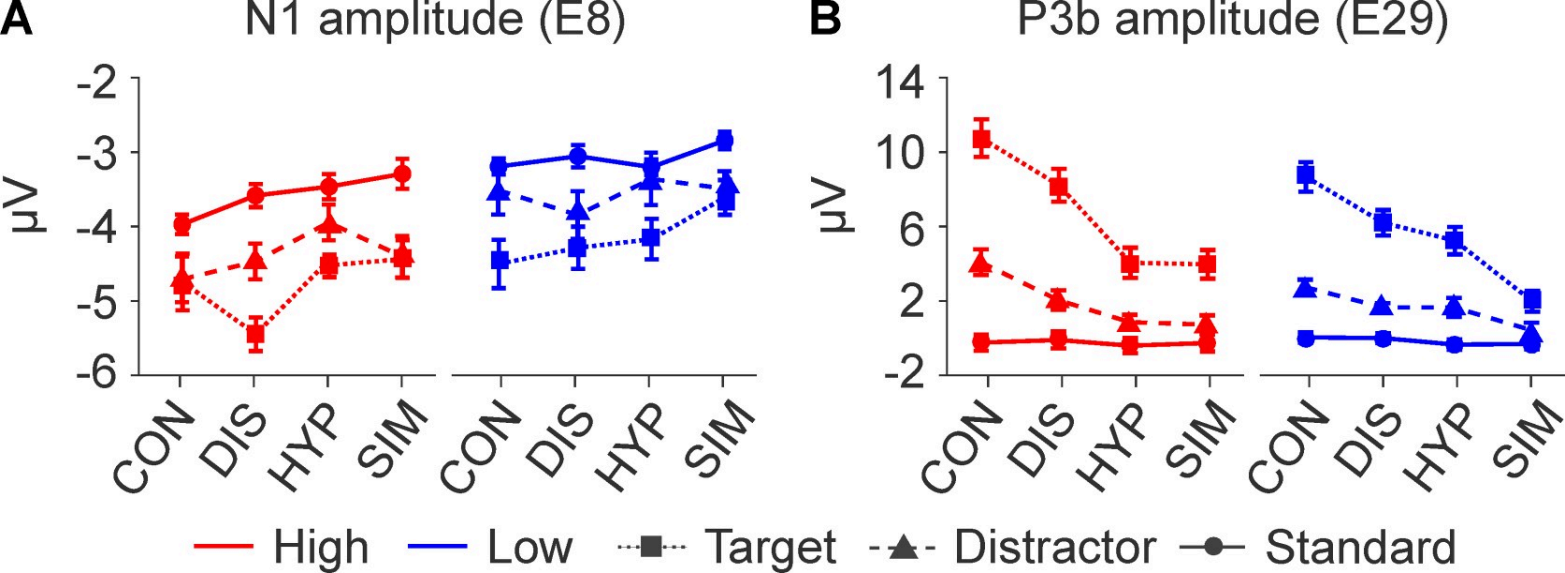
N1 and P3b amplitudes (mean ± within-subject standard error) at electrode E8 (Fz) and E29 (Pz), respectively, of high (red) and low (blue) susceptible participants in response to target (dotted line), distractor (dashed), and standard (solid) stimuli in the four experimental conditions: Control (CON), distraction (DIS), hypnosis (HYP), and simulation (SIM).

*N1 component*. The three-factorial analysis of N1 amplitudes at the frontocentral electrode 8, closely matching the Fz electrode of the International 10–20 system, revealed a significant main effect for Condition (*F*_2.5, 119.4_ = 3.8, *p* = .02, η_p_^2^ = 0.08). Post-hoc tests revealed a significant difference in N1 amplitude between DIS vs. SIM (*M = −4*.*1 vs*. *−3*.*7*, *t* 138 = –2.7, *p =* .05, Bonferroni adjusted). There was also a significant main effect for Stimulus-Type (*F*_1.7, 78.6_ = 35.4, *p* < .001, η_p_^2^ = 0.4); N1 amplitudes were largest for target stimuli (*M = −4*.*5)*, followed by the distractor (*M = −4*.*0)*, and standard (*M = −3*.*3*), see Fig 3A. No further main or interaction effect was significant.

*P3b component*. The ANOVA for repeated measures for P3b amplitude at electrode 29 (about Pz within the 10–20 system) revealed no significant between-subjects effect of both susceptibility groups. However, there was a highly significant main effect for factor Condition (*F*_2.4, 109.4_ = 41.58, *p* = < .001, η_p_^2^ = .48) with largest P3b amplitudes during CON, followed by DIS, HYP, and SIM (see Fig 3B). Post-hoc tests between CON and DIS, HYP, SIM were significant as well as between DIS and HYP, SIM (for details of post-hoc tests see S1-5 Table of S1 File). However, there was no significant difference of P3b amplitude between HYP and SIM. There was a significant interaction between factors Condition and Susceptibility (*F*_2.4, 109.4_ = 3.2, *p* = .04, η_p_^2^ = .06) with slightly lower P3b amplitudes of high susceptibles than of low susceptibles across all conditions and a huge significant effect for factor Stimulus-Type (*F*_1.2, 53.5_ = 99.3, *p* < .001, η_p_^2^ = .68). Post-hoc tests of factor Stimulus-Type confirmend larger P3b amplitudes for target stimuli, followed by distractor and standard stimuli (for details of post-hoc tests, see S1-6 Table of S1 File). There was no significant effect for the interaction between factor Stimulus-Type and Susceptibility but a significant interaction between factors Condition and Stimulus-Type (*F*_4.4, 204.3_ = 23.82, *p* < .001, η_p_^2^ = .34, see S1-7 Table of S1 File for post-hoc tests). The 3-way interaction Condition by Stimulus-Type by Susceptibility failed significance. Concerning the topography of P3b amplitude in response to the stimuli, the analysis revealed the well-known topographical distribution of P3b mainly above posterior areas of the brain with larger extension of P3b activity during the CON condition and flaring spreads for the DIS, HYP, and SIM conditions. Similar topographies cannot be seen for the standard and distractor stimuli.

#### Topography-by-time-cluster-analyses

*N1 component*. The three-factorial TTCA analysis of N1 amplitudes within the time window from 70 to 130 ms post-stimulus only revealed a significant main effect for Stimulus-Type (S1-1 Fig and S1-8 Table of S1 File). Importantly, and contrary to the single electrode analysis at E8, there was no significant difference in N1 amplitudes between conditions.

*P3 component*. The corresponding three-factorial analysis of scalp P3b amplitudes within the window of 320 to 470 ms post-stimulus provided a significant main effect for Stimulus-Type (S1-2B Fig and S1-9 Table of S1 File). P3b amplitudes were largest for the target stimulus, followed by the distractor, and smallest for the standard (S1-2A Fig of S1 File) corresponding to the well-replicated oddball effect. Additionally, we observed a significant main effect for Condition (S1-3 Fig and S1-10 Table of S1 File) and a significant two-way interaction of Susceptibility and Condition (S1-4 Fig and S1-11 Table of S1 File). Most importantly, we found a significant two-way interaction of Condition and Stimulus-type (S1-5 Fig and S1-12 Table of S1 File) indicating that P3b amplitude differences between the four conditions varied by Stimulus-Type. To disentangle the Condition by Stimulus-Type interaction, a simple effects analysis was conducted. The corresponding scalp-time SPM results for relevant t-Contrasts (CON vs. DIS, CON vs. HYP, CON vs. S1 M, DIS vs. HYP, and HYP vs. SIM) separated by Stimulus-Type are shown in Fig 1B. The P3b amplitudes of the target were significantly larger in CON vs. DIS, HYP and SIM. There was no significant main effect for Susceptibility. Likewise, the two-way interaction of Susceptibility by Stimulus-Type, and the three-way interaction of Susceptibility by Condition by Stimulus -Type were also not significant.

### Source analysis

Across subjects, the multiple sparse prior (MSP) based source reconstruction approach explained on average 86.0% (Min–Max: 31.2–97.1%) of scalp ERP variance in the interval from −100 to 600 ms across the four conditions and three stimulus types. Since the sensor analysis (TTCA) did not reveal any condition differences for the auditory N1 component, but for the P3b, subsequent statistical analyses were limited to the P3b window.

#### P3b sources

**Fig 4:**
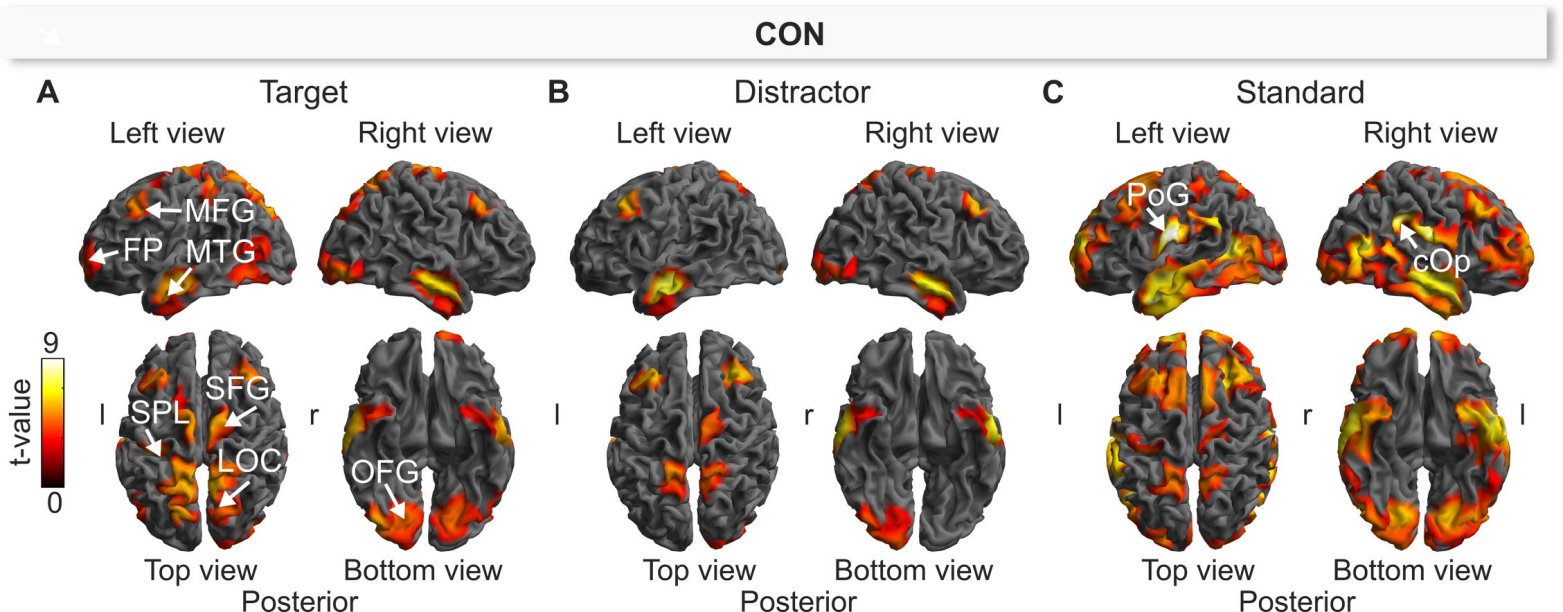
Source-level effects of the auditory Three-Stimulus-Oddball paradigm during the P3b window (320–470 ms) in control (CON) for the target (A), distractor (B), and standard (C). The cortical images show sources that were significantly different from zero (one-sample *t*-test). The labeled brain structures refer to the cluster peaks. l = left; r = right; MTG = middle temporal gyrus; FP = frontal pole; SPL = superior parietal lobule LOC = lateral occipital cortex; OFG = occipital fusiform gyrus; SFG = superior frontal gyrus; PoG = postcentral gyrus; cOP = central opercular cortex. The summary statistic images of the cortical mesh were thresholded at uncorrected *p* = .001 with FWE correction at cluster-level, *p* = .05, based on random field theory.

Fig 4 illustrates sources that significantly contributed to the scalp potential within the P3b window for the CON condition displayed separately for processing of targets, distractors, and standards. In particular, the deep red to yellow regions in Fig 4 represent source clusters whose activation was significantly different from zero within the P3b window (S1-14 Table of S1 File). For all three stimuli, the analyses revealed a widely distributed network of sources including structures in the mediofrontal/superiofrontal gyrus (MFG/SFG), middle temporal gyrus (MTG), occipital fusiform gyrus (OFG), and laterooccipital cortex (LOC), whereas activations in the frontal pole (FP) and superior parietal lobule (SPL) only contributed to the processing of target and standard stimuli (Fig 4A and 4C), but not to processing of distractors (Fig 4B). In addition, the processing of standard stimuli also included structures in the central operculum (cOp), the postcentral gyrus (PoG), and the frontal pole (FP). According to Fig 4, the most source clusters–both in number and in spatial extent–contributed to the standard stimuli, which is most likely due to the fact that these stimuli were presented in 80% of all trials in each of the four experimental conditions and therefore induced the most consistent and least variant activations across all participants compared to the two rarely presented target and distractor stimuli that were presented in only 10% of trials each.

**Fig 5:**
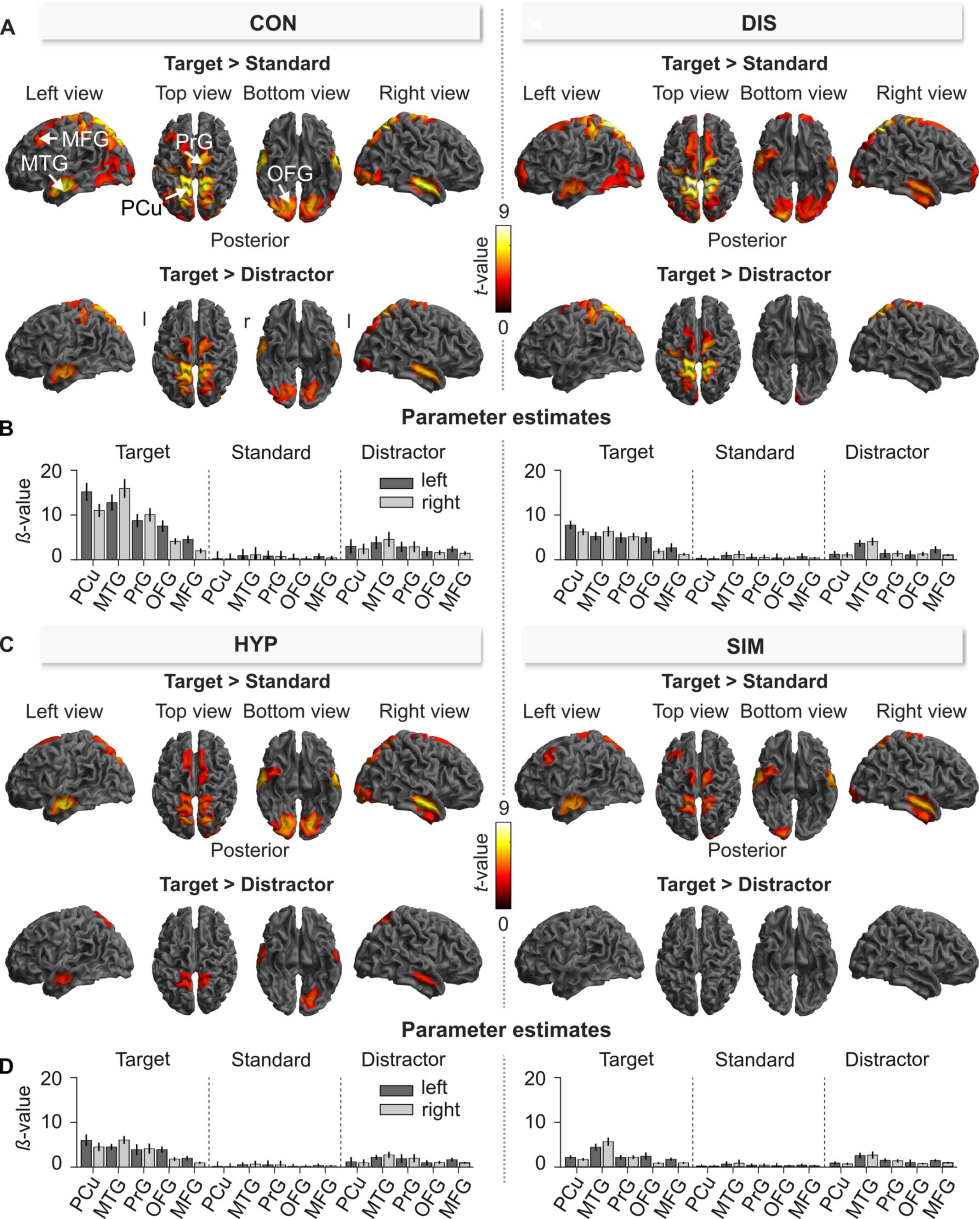
Source-level effects of the auditory Three-Stimulus-Oddball paradigm during the P3b window (320–470 ms) in the control (CON), distraction (DIS), hypnosis (HYP), and simulation (SIM) condition. **(A/C)** Statistical comparison (*t*-contrast) of Target > Standard (top row) and Target > Distractor (bottom row) for source activities within the P3b window in the CON, DIS, HYP and SIM. The cortical images show sources that were significantly more activated following processing of target as compared to standard stimulus. The labeled brain structures refer to the cluster peaks. l = left; r = right; MFG = middle frontal gyrus; MTG = middle temporal gyrus; PCu = precuneous; OFG = occipital fusiform gyrus; PrG = precentral gyrus. The summary statistic images of the cortical mesh were thresholded at uncorrected *p* = .001 with FWE correction at cluster-level, *p* = .05, based on random field theory. **(B/D)** Parameter estimates (*β*-value, 90% C.I.) of target, distractor and standard in CON, DIS, HYP, and SIM for sources located within cluster peaks of the left and right hemisphere (see Fig 5A, top row) where sources in question are located. Values of source strength are expressed as root mean square (RMS, in arbitrary units). Grey rectangle marks the P3b window.

Since the P3b component is differentially influenced by stimulus-type, we contrasted the processing of target stimuli against standard stimuli and distractor stimuli to specifically examine target related activity of the P3b at the source-level. Fig 5A and 5C depicts the source clusters that showed significantly larger source activities for targets compared to standards (top row) and for targets compared to distractors (bottom row) in each of the four experimental conditions:

*Target vs*. *standard*. In the CON condition, the contrast Target > Standard revealed a very similar network of multiple sources as described above in Fig 4A for the target indicating that all of these sources showed significantly stronger activations during target processing than of standards (Fig 5A, top left). These differential activations are greatest under CON, and gradually decreased from DIS (Fig 5A, top right) to HYP to SIM (Fig 5C, top left and right), both in spatial extent and source strength. This is also nicely reflected in the bar plots of beta weights for the respective source clusters depicted separately for each stimulus type in each condition (Fig 5B and 5D). For statistical results see S1-15 Table of S1 File.

*Target vs*. *distractor*. In the CON condition, the contrast Target > Distractor revealed a less extensive network compared to target vs. standard stimuli (Fig 5A, bottom left). This may be due to the fact that targets and distractors only differ with respect to task relevancy, while targets and standards differ in task relevancy and probability of occurrence. In CON, targets compared to distractors elicited stronger source activities within the parietal cortex (PCu), the precentral gyrus (PrG), the mediotemporal gyrus (MTG) and the occipital fusiform gyrus (OFG). Interestingly, the DIS condition showed no differences in source activities within the MTG or the OFG (Fig 5A, bottom right). During HYP, differences between target and distractor processing were even less pronounced with small clusters in the MTG, OFG, and the parietal cortex (PCu) whereas in SIM no differences were observed between target and distractor processing (Fig 5C, bottom). For statistical results see S1-16 Table of S1 File.

#### Experimental condition effects

**Fig 6:**
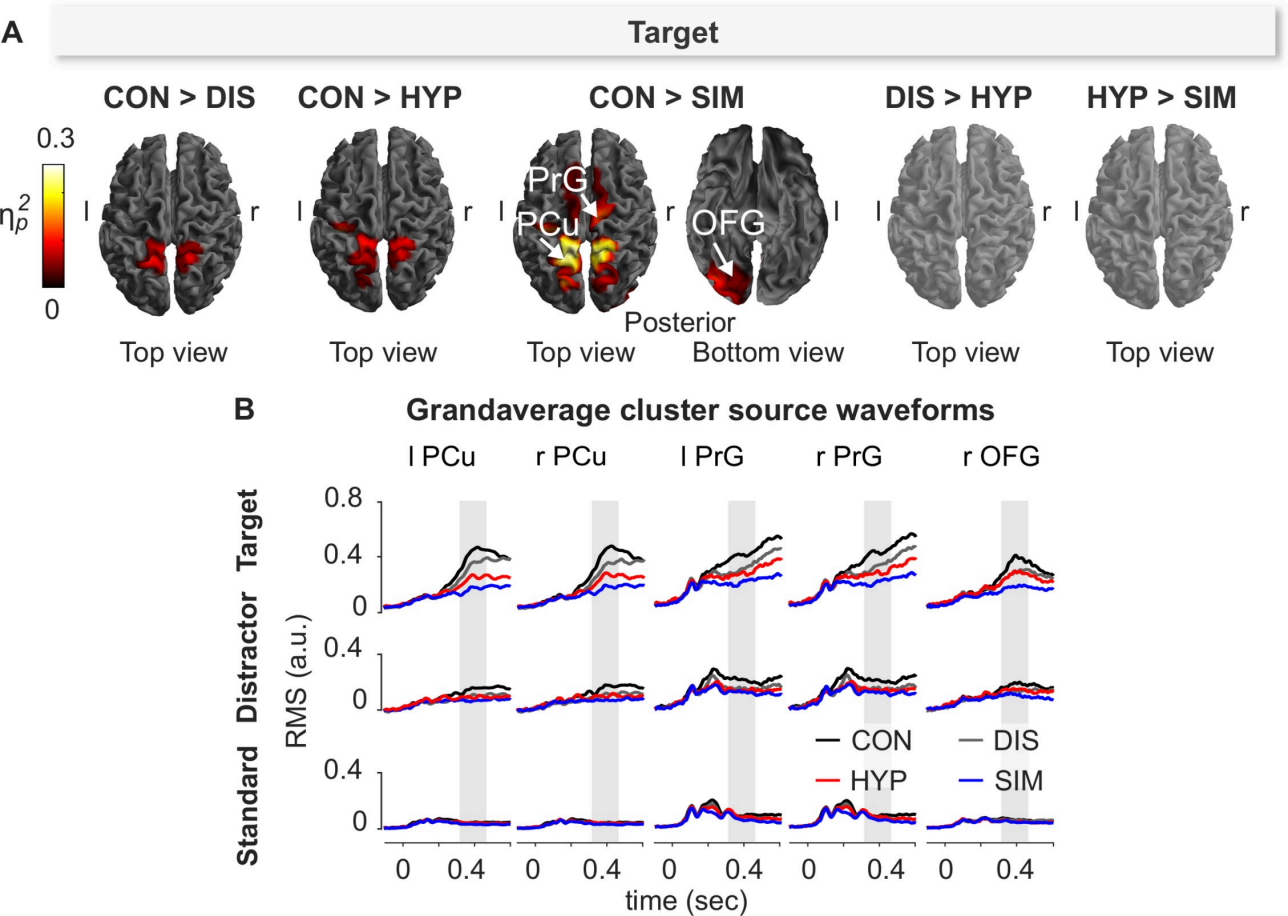
Effect size ($\eta_{p}^{2}$) of statistical comparison (*t*-contrast) of control (CON) vs. distraction (DIS), CON vs. hypnosis (HYP), and CON vs. simulation (SIM) for processing of target stimulus. The comparisons of DIS vs. SIM and HYP vs. SIM were not significant. **(A)** The cortical mesh images show sources that were significantly less activated in HYP, DIS and SIM compared to CON following processing of target. PCu = precuneous; PrG = precentral gyrus; OFG = occipital fusiform gyrus. The summary statistic images of the cortical mesh were thresholded at uncorrected *p* = .001 with FWE correction at cluster-level, *p* = .0045 (one-sided, Bonferroni), based on random field theory. r = right, l = left. **(B)** The grandaverage cluster source waveforms (across subjects, *n* = 48) are based on the average of source waveforms within the respective clusters found in Fig 5A (CON > SIM) for the three Stimulus-Types in CON (black), HYP (red), DIS (grey), and SIM (blue). Source strength is expressed as root mean square (RMS, in arbitrary units). Grey rectangle marks the P3b window (320–470 ms).

Since target processing was significantly reduced during the experimental conditions DIS, HYP and SIM as compared to CON at sensor-level, we examined where these P3b differences were reflected at the source-level. Fig 6A depicts the respective effect sizes ($\eta_{p}^{2}$) for the significant source clusters (S1-17 Table of S1 File). During DIS compared to CON, target processing was associated with significantly less activation in the parietal cortex including the lateral occipital cortex (LOC) and the superior parietal lobule (SPL). Likewise, target processing under HYP was significantly reduced in the parietal cortex (SPL) compared to CON. The largest differential effects were observed during SIM compared to CON; here target source activities were significantly smaller within the parietal cortex (LOC, PCu), the inferior division of the LOC (OFG), and the precentral gyrus (PrG) including the supplementary motor area (SMA). Interestingly, there were no significant differences between DIS > HYP as well as HYP > SIM for target processing. Contrary to sensor-level results, we did not observe any significant difference of source activities between the experimental conditions HYP, DIS, SIM and CON for distractor and standard stimuli. The grandaverage source waveforms of the significant cluster peaks are illustrated in Fig 6B.

Remarkably, source activities of occipital (OFG) and parietal regions (PCu) peaked in the P3b time window during target processing whereas precentral (PrG) source clusters including the SMA peaked ~600 ms poststimulus (Fig 6B) which corresponds approximately to the mean target response time in each of the four conditions (see Fig 1E). Thus, the timing and location of these source clusters indicate different cognitive processes.

## Discussion

### Behavioral data

The majority of participants implemented our request to adopt the most supportive, active, and positive attitude possible during all experimental sections and took an active, curious role in getting hypnotized during the HYP condition. They also realized all task instructions very compliantly and were very engaged in pretending being hypnotized by displaying a series of behaviors which they remembered from previous individual experiences with hypnosis (f.e. during the preceding HGSHS testing or the HYP condition that might have preceded the SIM condition according to the balancing procedure of the whole experiment for half the participants) and considered typical for being hypnotized and best able to convience the external, unknown observer that they got hypnotized. Furthermore, in order to strengthen their efforts in simulating hypnosis, a financial reward was announced for the three most successful role players. Participants also were instructed very detailed about the CON condition where they should shift their attention from the stimuli to a film and remember as many details of the film as possible (DIS condition).

For example, in all four conditions, most of the participants only pressed the response key when targets were presented and only very rarely when non-targets were presented. In CON, participants revealed very high hit rates (low susceptibles: 94%, high susceptibles: 92%) while the hit rates were markedly reduced during HYP (lows: 65%, highs: 30%, see Fig 1A). Thus, there is clear evidence that the suggestions of deafness affected participants’ behavior and significantly reduced their responses to target stimuli. But this result does not necessarily indicate that the perception of non-targets was abolished. They must have been categorized at least as non-targets, since otherwise the target could not have become discriminated as response-relevant stimulus in almost two thirds of target trials by low, and one third of target trials by high susceptibles. In regard to earlier studies, hit rates of high susceptibles during HYP and SIM of the present study replicate the observations of the Erickson (1938, 1939), Dynes (1932) and Barber et al. (1964) and the study by Crawford and colleagues and Spanos and coworkers (1982). Compared to CON, these effects of hypnotic suggestion were also retrospectively expressed in reduced tone loudness. Concerning target response time, HYP significantly slowed down participants’ responses compared to CON. This prolonged response time seems to reflect costs owing to the organization of key presses in a condition of conflict where participants are challenged to react to targets, but at the same time complying to the suggestions not to perceive the targets due to deafness.

While simulating hypnosis (SIM), we found rather similar effects of reduced hit rates (lows: 32%, highs: 25%) as during HYP but no significant effect on tone loudness in high and low susceptibles. It is important to note that participants were asked they should not simulate but report the loudness as it was really felt during SIM. Similar to HYP, response time was also significantly delayed while participants simulated hypnosis.

Concerning the DIS condition, target hit rates of low and high susceptible participants were fairly similar around 87% and 80%, respectively, suggesting that distraction did not much affect participants’ behavior. Also, distraction caused almost no change in tone loudness for both groups. Furthermore, processing and storing information related to the film presented during the DIS condition seems to slow down the reaction times less than hypnotic suggestions and caused only a slight delay of reaction time compared to the CON condition.

In summary, the behavioral observations indicate that hypnotic deafness and simulation of deafness led to comparable effects for hit rates and response times but not for tone loudness. While hits and response time were obtained within the oddball presentations, loudness was assessed at the end of the oddball paradigm in each condition. Whether this difference of loudness between HYP and SIM indicates that the suggestion during SIM was ineffective or a consequence of different demand characteristics of both conditions [64, 65] is hard to decide since debriefing with participants at the end of the experiment did not provide clear information. Yet, the observed behavioral differences of low and high susceptibles in the HYP and SIM vs. DIS condition clearly specifies that hypnotic deafness or simulation of deafness cannot be based on comparable mechanisms. Likewise, changes in attention seem not being sufficient to explain the effects of hypnosis. In regard to the behavioral effects of HYP and SIM, the old role-playing concept [66, 67] or the expectation theory of hypnosis [68] might provide a sufficient explanation for both conditions.

### Sensor analysis

Concerning the neural brain processes during the four experimental conditions, the present study is the first one that used a three-stimulus oddball paradigm for the investigation of dense array brain electrical, event-related potentials of high and low susceptible participants as neural signatures of auditory stimulus processing. Earlier studies, f.e., by Barabasz and coworkers [29] only investigated the P300 amplitude in response to a one-stimulus paradigm and identified an earlier component whose functional relationship to deafness or other aspects of stimulus processing were not outlined clearly [69]. Since the present study focused on the sources of P300 amplitude, we forego the discussion of other stimulus modalities and refer to a similar study in which we examined a comparable design for a visual three-stimulus oddball [70], and only focus on some interpretations of the present study. In this independent study [70], participants completed a visual oddball paradigm composed of standard, distractor, and target stimuli during a hypnosis (HYP) and a control (CON) condition and were suggested that a wooden board in front of their eyes would obstruct their view of the screen. In contrast to the present study, participants were asked to count the rare visual targets presented on a video screen. The results show that participants’ counting accuracy was significantly impaired during HYP compared to CON. Earlier brain responses at 80 to 170 ms post-stimulus (i.e., N1 and P2) revealed no amplitude differences between CON and HYP at sensor-level. In contrast, P3b amplitudes in response to target stimuli were significantly reduced in HYP compared to CON whereas P3b amplitudes in response to standard and distractor stimuli remained almost unchanged.

Analysis of ERP amplitudes of the present study mainly addressed two late ERP components that are functionally related to cognitive processes of stimulus information, i.e., the N1 and P3b components [31, 32, 71]. Analyses of N1 amplitudes of the present study support all topographical and functional aspects of this component as described above and revealed a frontal topography. Its amplitudes varied significantly as a function of Stimulus-Type with decreasing magnitudes from targets to standards (S1-1 Fig and S1-3 Table of S1 File). However, N1 amplitude was not significantly affected by hypnosis, which is in line with our previous study using a visual three-stimulus oddball paradigm [10], and series of earlier studies of our group [37, 40–43] and a study by Bromm and coworkers [72] investigating the effects of hypnosis on the perception of pain. Thus, these results indicate that the stimuli were preattentively processed and focused equally well by low and high susceptibles in all four experimental conditions. In our opinion, this precludes arguments that artifacts or trivial meditators such as defocusing attention from the tones or decreasing subjects’ activation etc. might represent the main reasons for the effects in participants’ behavioral responses or subsequent brain-electrical processes of this study.

Supporting the functional relationships of P3b amplitudes as outlined above, P3b results of the present study varied according to the task functions of stimuli and their respective stimulus probabilities. It thus replicated the classic impact of these cognitive functions on P3b amplitude: Largest P3b amplitudes and P3b cluster activities were observed in response to rare target stimuli followed by substantially smaller P3b amplitudes to rare distractors and almost no P3b activity was seen to standard stimuli. Significant differences of P3b activity were not observed between low and high suggestible participants in all experimental conditions which is in line with our previous study of the visual domain [10]. However, P3b activity to target stimuli was significantly affected by the distracting presentation of a film (DIS), the hypnotic suggestions of deafness (HYP), and the simulation of hypnosis (SIM) with largest P3b amplitudes in CON, followed by gradually smaller amplitudes in DIS, HYP, and SIM (see Fig 2A).

In regard to the topography of P3b amplitude to targets and partly also to distractor stimuli, it is to assume that the activity of electrode E29 and the cluster around this electrode in the center of the parietal cortex significantly reflect the activity of the precuneus, for which several studies corroborated a putative role in stimulus-related attention and the mapping of responses to task-related stimuli [73]. The change of the topography of this centroparietal cluster along with the change of functional properties of the three stimuli confirms many previous findings that P3b activity is closely linked to stimulus probability and the task relevancy of stimuli [74]. Obviously, this structure loses most of its activation during HYP as compared to CON and DIS, and completely disappeared during the SIM condition in response to target stimuli. This activation is also much less present in response to distractor in the CON, DIS, SIM and HYP conditions and consistently very small during frequent and task irrelevant stimulation (standards),

### Source analysis

The source analysis of the P3b component revealed several large source clusters within the cerebral cortex for all three stimuli (see Fig 4): [1] A large source cluster was located in the middle (MTG) temporal gyri including the inferior temporal gyri (ITG) of both hemispheres that together are known to assist the processing of complex auditory stimuli and the differentiation among auditory stimulus patterns [75–77]. [2] The primary occipital cortex (LOC, including the occipital fusyfom gyrus OFG) of both hemispheres whose neurons are critical for the processing of auditory frequencies [78]. [3] Large clusters in the superior parietal lobule and the post central gyri (PCG) of both hemispheres that house the precuneus cortex (PCu), which is especially critical for attentional focusing and for a wide spectrum of highly integrated tasks including audio-spatial imagery, episodic memory retrieval, and self-processing operations like first-person perspective taking and the experience of agency [73]. It was also discussed as a network of self-consciousness and self-related mental representations during rest and in many special cognitive states that are commonly summarized under the concept of altered states of consciousnes such as sleep or anesthesia [73]. [4] A large cluster in the superior frontal gyrus (SFG), also called precentral gyrus PrG [79] that is juxtapositional to the supplementary motor area (SMA) and large clusters in the frontal pole (FP), the latter in the case of target and standard stimuli, that are especially involved in motor programming [79] and supervision [80, 81]. These source patterns replicate earlier source studies of the auditory P3b which also identified similar sources like the present study using either fMRI methods [78, 82–86] or ERP methods and the sLORETA source approach [87]. Our findings also support that the P3b amplitude is constituted by varying sources, depending on the modality and complexity of stimuli and the tasks to which they are assigned to [88].

Fig 5 indicates that target processing induced the strongest activation compared to the processing of distractor and standard stimuli in the occipital fusiform gyrus (OFG), precuneus (PCu), middle temporal gyrus (MTG), precentral gyrus (PrG) and middle frontal gyrus (MFG). The reason for this is associated with the special task relevancy of the targets that require proper frequency discrimination within the OFG and MTG, strong attentional focusing by activity in the PrG and PCu and proper response preparation in the SMA as part of the MFG. The target source activities of the PCu cluster were significantly lower during HYP vs. CON and likely reflect the fact that only a few numbers of targets received proper attentional focus followed by proper key presses (Fig 6A). During SIM compared to CON, we also observed lower source activity in the right OFG in response to targets signaling stimulus discrimination, and lower source activities in the PrG/SMA indicating less response preparation for the few hits during SIM. This is clearly demonstrated by the time course of source activation during target processing as outlined in Fig 6B. PrG/SMA activation remains high beyond the P3b window, likely indicating activity associated with the motor-related bereitschaftspotential (readiness potential) for programming of key presses [89, 90]. In contrast, OFG and PCu activity already faded within the P3b window. During the processing of distractor and standard stimuli these patterns were not present in all five sources and constantly reflected very low source activation since these stimuli had to be ignored.

In summary, the study shows that the processing of stimuli during the four experimental conditions is associated with different P3b-related numbers of active sources and magnitudes of source activity. What we consider as rather surprising is the fact, that obviously only a small number of sources that organize the processing of auditory stimuli are critical for the differences of P3b amplitude and cluster activities in response to target stimuli between the control condition and distraction, hypnosis, and simulation of hypnosis conditions. These sources all are part of central auditory processing areas that either control the focus of attention, the discrimination of auditory stimuli, and the organization of behavioral responses to targets.

## Conclusion

Related to research question [1], the present study supports earlier observations that the perception of acoustic stimuli can be significantly affected by hypnotic deafness suggestions. Behavioural responses (loudness, *d-*prime) and neural activities (P3b and its underlying neural sources) get significantly reduced while participants were hypnotized and suggested not to hear rare and frequent tones. However, perception of stimuli was not completely abolished because several targets still were detected correctly. Since equivalent responses for the processing of irrelevant distractor and standard stimuli are not provided by the paradigm, confirmative evidence for similar processing of these stimuli can be derived from the N1 amplitude as neural signature of preattentive stimulus discrimination. The magnitude of the N1 component did not significantly differ from each other in all four conditions.

In regard to research question [2], i.e., whether behavioural and neural effects of HYP differ from SIM, the results demonstrate similar reductions of *d-*prime during SIM and HYP but no change of loudness. Thus, the instruction to pretend not to hear the tones was compliantly realized for key presses but led to no changes of the experienced loudness. We think that one reason for the failure of loudness reduction relates to the fact that that participants were required to report the perceived but not the simulated loudness. If simulated deafness lowers the task relevancy of the target then − according to Verleger’s suggestion [46] that P3b magnitude reflects the association strength of a stimulus to its task − P3b amplitude should be reduced. Concerning DIS, we did not observe reductions of hit rates and loudness and only small reductions of P3b activities in response to targets and distractors. This supports our earlier investigations [40–42] that attentional processes alone are not sufficient to explain the effects of hypnosis. With respect to susceptibility [i.e., research question 3], the effects of the hypnotic suggestion of deafness were larger in high than in low susceptibles in each of the three behavioral parameters (*d-*prime, response time, loudness) whereas no significant group differences were observed in neural responses of N1 and P3b components and of simulated deafness. In contrast, simulated deafness only led to longer response times in high but not in low susceptibles. Finally, concerning research question [4], our study of ERP activity provides interesting insight into possible cognitive mechanisms that differ hypnosis from simulation of hypnosis. Topographical distribution of P3b activities and Topography-by-Time-Cluster-Analyses of the amplitude show differences of the time course and spatial extent of activation in parietal brain areas in response to target stimuli (Fig 3B and 3C). Obviously, HYP is associated with larger P3b activities in temporoparietal structures than SIM whose significant differences are expressed in later periods of the P3b window. According to the cognitive functions of this temporoparietal cluster, this difference likely reflects that participants allocated more attentional resources to the stimuli during HYP than during SIM and seem to cogitate how the conflict between the requirement to respond to but not to perceive the stimulus could be solved. This conflict seems not present during SIM since here participants simulated not to hear the target and subsequently pressed the key less frequently. Additional information is provided by reduced activity of PrG/SMA sources (Fig 6) that are involved in P3b generation and relevant for stimulus-response mapping [46].

For the synopsis of the entire study, our data confirm that deafness suggestions during hypnosis lead to a significant change of auditory perception. However, we also demonstrate that complete deafness is hard to achieve since most of our participants responded to tones as indicated by non-zero hit rates and less neural responses to tones. Therefore, the term ‘deafness’ may be misleading and should better be replaced by ‘hypoacusis’. With the exception of loudness, this was also seen when deafness was simulated during SIM.

Although doubts about the usefulness of neuroscientific methods for elucidating the mechanisms of hypnosis have occasionally been articulated in the past [91], our study supports the results and conclusion of a recent international conference on the importance of neuroscience for the study of hypnosis that the investigation of neuroscientific processes provides insight into mechanisms and processes that would remain uncovered by pure behavioral or cognitive methods and measures [92].

## Supporting information

S1 File Additional methods and results. (PDF)

S2 File Wording of experimental instructions and tests. (PDF)
