# Supplementary material for: Suggested deafness during hypnosis and simulation of hypnosis compared to a distraction and control condition: A study on subjective experience and cortical brain responses
Source: PLoS One. 2020 Oct 29;15(10):e0240832. doi: 10.1371/journal.pone.0240832 (PMC7595429; doi:10.1371/journal.pone.0240832)
Supplement: S1 File — (PDF) [file pone.0240832.s001.pdf]

Supplemental information 1 to the article:

Suggested deafness during hypnosis and simulation of hypnosis compared to a distraction and control condition: A study on subjective experience and cortical brain responses.

Marcel Franz[1], Barbara Schmidt[1], Holger Hecht[1], Ewald Naumann[2] and Wolfgang H.R. Miltner[1,3]

[1]Department of Clinical Psychology, Friedrich Schiller University Jena, Jena, Germany

[2]Institute of Psychology, University of Trier, Trier, Germany

[3]Medical School of Friedrich Schiller University Jena, Jena, Germany

METHODS

In the following section, we outline how the three factorial (Condition x Stimulus-Type x Susceptibility-Group) nature of our design was modeled and statistically tested in SPM12. We followed the partitioned error approach as outlined in the SPM wikibook (SPM, 2018). The subject-specific set of 4-by-3 GIFTI images (condition: c1 = hypnosis, c2 = control, c3 = distraction, c4 = simulation; stimulus type: s1 = target, s2 = standard, s3 = distractor) was arranged in the following order: c1s1 c1s2 c1s3 c2s1 c2s2 c2s3 c3s1 c3s2 c3s3 c4s1 c4s2 c4s3. This set was first transformed into four sets of contrast images using the spm_mesh_calc.m function and the respective contrast weights C of **Table S1-1**:

Table S1-1. Effects of interest and the corresponding contrast weights to calculate 1st level contrast images using the spm_mesh_calc function.

**Effect of interest:**	**Contrast Image Set**	**1st level Contrast weights**
E1 = ME: Group	1	$C_1 = [1\ 1\ 1\ 1\ 1\ 1\ 1\ 1\ 1\ 1\ 1\ 1]$
E2 = ME: Condition; E3 = INT: Group x Condition	2	$C_2 = \begin{bmatrix} 1\ 1\ 1\ \text{-}1\ \text{-}1\ \text{-}1\ 0\ 0\ 0\ 0\ 0\ 0 \\ 0\ 0\ 0\ 1\ 1\ 1\ \text{-}1\ \text{-}1\ \text{-}1\ 0\ 0\ 0 \\ 0\ 0\ 0\ 0\ 0\ 0\ 1\ 1\ 1\ \text{-}1\ \text{-}1\ \text{-}1 \end{bmatrix}$
E4 = ME: Stimulus type; E5 = INT: Group x Stimulus−Type	3	$C_3 = \begin{bmatrix} 1\ \text{-}1\ 0\ 1\ \text{-}1\ 0\ 1\ \text{-}1\ 0\ 1\ \text{-}1\ 0 \\ 0\ 1\ \text{-}1\ 0\ 1\ \text{-}1\ 0\ 1\ \text{-}1\ 0\ 1\ \text{-}1 \end{bmatrix}$
E6 = INT: Condition x Stimulus type; E7 = INT: Group x Condition x Stimulus type	4	$C_4 = \begin{bmatrix} 1\ \text{-}1\ 0\ \text{-}1\ 1\ 0\ 0\ 0\ 0\ 0\ 0\ 0 \\ 0\ 1\ \text{-}1\ 0\ \text{-}1\ 1\ 0\ 0\ 0\ 0\ 0\ 0 \\ 0\ 0\ 0\ 1\ \text{-}1\ 0\ \text{-}1\ 1\ 0\ 0\ 0\ 0 \\ 0\ 0\ 0\ 0\ 1\ \text{-}1\ 0\ \text{-}1\ 1\ 0\ 0\ 0 \\ 0\ 0\ 0\ 0\ 0\ 0\ 1\ \text{-}1\ 0\ \text{-}1\ 1\ 0 \\ 0\ 0\ 0\ 0\ 0\ 0\ 0\ 1\ \text{-}1\ 0\ \text{-}1\ 1 \end{bmatrix}$

E = effect, ME = main effect, INT = interaction

Then, for each set of contrast images, two General Linear Models (GLM) were specified and estimated to test for effects of interest. The respective factorial design specifications and 2nd level contrasts are summarized in **Table S1-2**.

Table S1-2. Factorial design specification.

**Effect of interest:**	**Contrast Image Set[1]**	**Factorial Design**	**2nd level Contrast Weights**
ME: Group	1	Two sample t-test[a,c]	F-contrast $[1 -1]$
ME: Condition	2	One-way ANOVA[b,c] (3 levels)	F-contrast $\begin{bmatrix}1&0&0\\0&1&0\\0&0&1\end{bmatrix}$
INT: Group x Condition	2	One-way ANOVA[a,c] (6 levels , 3 for each group)	F-contrast $\begin{bmatrix}1&0&0&-1&0&0\\0&1&0&0&-1&0\\0&0&1&0&0&-1\end{bmatrix}$
ME: Stimulus type	3	Two sample t-test[b,c]	F-contrast $\begin{bmatrix}1&0\\0&1\end{bmatrix}$
INT: Group x Stimulus type	3	One-way ANOVA (4 levels, 2 for each group)[a,c]	F-contrast $\begin{bmatrix}1&0&-1&0\\0&1&0&-1\end{bmatrix}$
INT: Condition x Stimulus type	4	One-way ANOVA (6 levels)[b,c]	F-contrast $\begin{bmatrix}1&0&0&0&0&0\\0&1&0&0&0&0\\0&0&1&0&0&0\\0&0&0&1&0&0\\0&0&0&0&1&0\\0&0&0&0&0&1\end{bmatrix}$
INT: Group x Condition x Stimulus type	4	One-way ANOVA (12 levels, 6 for each group)[a,c]	F-contrast $\begin{bmatrix}1&0&0&0&0&0&-1&0&0&0&0&0\\0&1&0&0&0&0&0&-1&0&0&0&0\\0&0&1&0&0&0&0&0&-1&0&0&0\\0&0&0&1&0&0&0&0&0&-1&0&0\\0&0&0&0&1&0&0&0&0&0&-1&0\\0&0&0&0&0&1&0&0&0&0&0&-1\end{bmatrix}$

[1] see Table S1-1

(a) independent measurements between levels (b) dependent measurements between levels (c) measurements in each level are assumed to have unequal variance

RESULTS

SENSOR-LEVEL ANALYSIS

Approach 1: Statistics on single-electrode analysis

Table S1-3. Post hoc comparisons – Condition. N1 amplitudes at electrode E8.

contrast	Mean difference	SE	df	*t*	*p*
CON − DIS	0.01	0.26	244.76	0.03	1.00
CON − HYP	-0.33	0.27	245.10	-1.24	1.00
CON − SIM	-0.43	0.26	244.97	-1.63	.52
DIS − HYP	-0.34	0.24	245.97	-1.40	.81
HYP − SIM	-0.10	0.24	255.76	-0.41	1.00

P-value adjusted (Bonferroni method) for comparing a family of 5
Degrees-of-freedom method: Satterthwaite
Results are averaged over the levels of: Group, Stimulus

Table S1-4. Post hoc comparisons – Stimulus. N1 amplitudes at electrode E8.

contrast	Mean difference	SE	df	*t*	*p*
Distractor − Standard	-0.64	0.20	344.23	-3.15	.005**
Distractor − Target	0.50	0.24	351.83	2.10	.11
Standard − Target	1.14	0.21	324.37	5.45	<.001***

P-value adjusted (Bonferroni method) for comparing a family of 3
Degrees-of-freedom method: satterthwaite
Results are averaged over the levels of: Group, Condition

Table S1-5. Post hoc comparisons – Condition. P3b amplitudes at electrode E29.

contrast	Mean difference	SE	df	*t*	*p*
CON − DIS	1.35	0.43	135.85	3.17	.01*
CON − HYP	2.52	0.42	144.91	5.98	<.001***
CON − SIM	3.28	0.40	135.17	8.13	<.001***
DIS − HYP	1.17	0.39	135.76	2.99	.016*
HYP − SIM	0.76	0.37	143.38	2.08	.196

P-value adjusted (Bonferroni method) for comparing a family of 5
Degrees-of-freedom method: Satterthwaite
Results are averaged over the levels of: Group, Stimulus

Table S1-6. Post hoc comparisons – Stimulus. P3b amplitudes at electrode E29.

contrast	Mean difference	SE	df	*t*	*p*
Distractor − Standard	1.99	.19	206.09	1.24	<.001***
Distractor − Target	−4.40	.42	257.94	−1.57	<.001***
Standard − Target	−6.39	.38	188.73	−16.86	<.001***

P-value adjusted (Bonferroni method) for comparing a family of 3
Degrees-of-freedom method: satterthwaite
Results are averaged over the levels of: Group, Condition

Table S1-7. Post hoc comparisons – Condition by Stimulus. P3b amplitudes at electrode E29.

stimulus	contrast	Mean difference	SE	df	*t*	*p*
Distractor	CON - DIS	1.57	0.54	79.43	2.92	.069
	CON - HYP	2.17	0.59	89.10	3.67	.006**
	CON - SIM	2.86	0.54	80.60	5.25	<.001***
	DIS - HYP	0.60	0.49	86.54	1.22	1
	HYP - SIM	0.69	0.50	87.95	1.38	1
Standard	CON - DIS	-0.05	0.19	86.49	-0.26	1
	CON - HYP	0.28	0.21	91.25	1.33	1
	CON - SIM	0.19	0.19	80.48	1.02	1
	DIS - HYP	0.33	0.18	89.55	1.78	1
	HYP - SIM	-0.09	0.17	84.50	-0.50	1
Target	CON - DIS	2.52	1.14	91.43	2.21	.445
	CON - HYP	5.10	1.10	89.49	4.65	<.001***
	CON - SIM	6.79	1.06	86.84	6.38	<.001***
	DIS - HYP	2.58	1.05	91.30	2.46	.235
	HYP - SIM	1.69	0.96	91.43	1.75	1

P-value adjusted (Bonferroni method) for comparing a family of 15

Degrees-of-freedom method: satterthwaite

Results are averaged over the levels of: Group

Approach 2: Statistics on Topography-by-Time-Cluster-Analyses

- ### N1 window (70–130 ms)

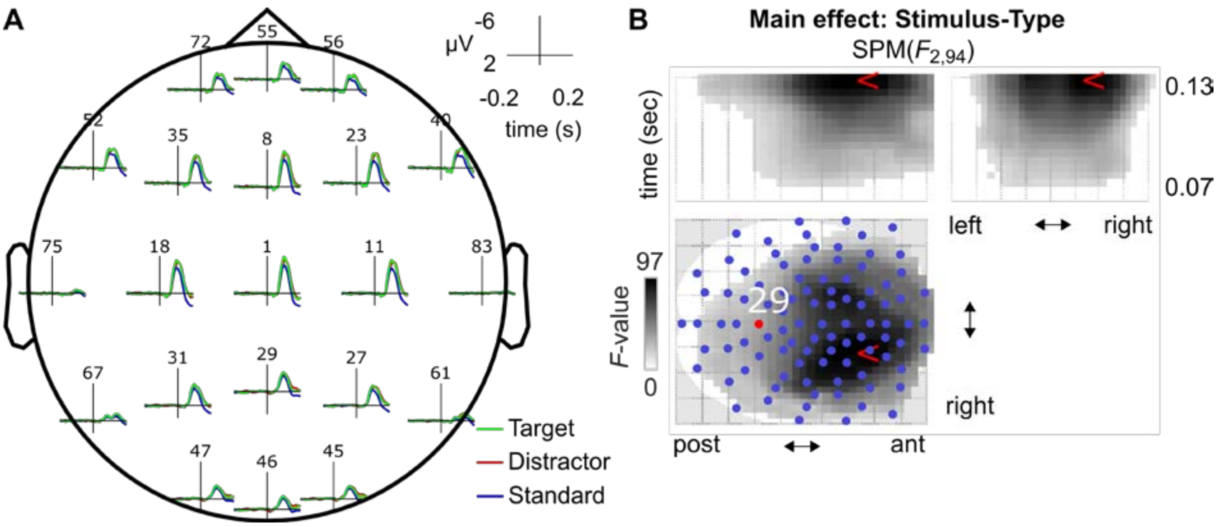

Figure S1-1. (A) Grandaverage N1 waveforms (pooled across subjects and conditions) for target (green), distractor (red), and standard (blue) stimulus type at selected electrode sites (21 out of 91 recorded). Data are referenced to linked mastoids. **(B)** Main effect of Stimulus-Type within N1 window (70−130 ms). A paired-sample *t*-test (within-subject) was performed and the results were assessed with F-contrast. The summary statistic scalp-time data volume was thresholded at p <.001 (uncorrected) with FWE correction at cluster-level, p <.05, based on random field theory. Blue dots mark electrode sites and the redarrows point to the global maximum. For details, see **Table S1-8**.

Table S1-8. Main effect of factor Stimulus-Type within N1 window (70−130 ms). Statistical results of scalp-time analysis.

**Cluster-level**		**Peak-level**				
$p_{FWE\text{-}corr}$	K_E	***F*-value**	$p_{uncorrected}$	**mm**	**mm**	**ms**
<.001	7064	97.26	<.001	21	24	128

Statistics: $p_{FWE\text{-}corr}$ = Family-wise-error corrected *p*-values adjusted for search volume. K_E = cluster with *K* elements. Height threshold: $F = 7.44$, $p = .001$ (uncorrected); df = (2, 94); Extent threshold: $k = 135$ bins; Smoothness FWHM = 32.2 47.3 22.2 mm mm ms; Expected bins per cluster <*k*> = 135.4; Search vol.: 1182575 = 12942 bins = 29.2 resels; Bin size: 4.2 5.4 4.0 mm mm ms; (resel = 370 bins).

- **P3b window (320−470 ms)**

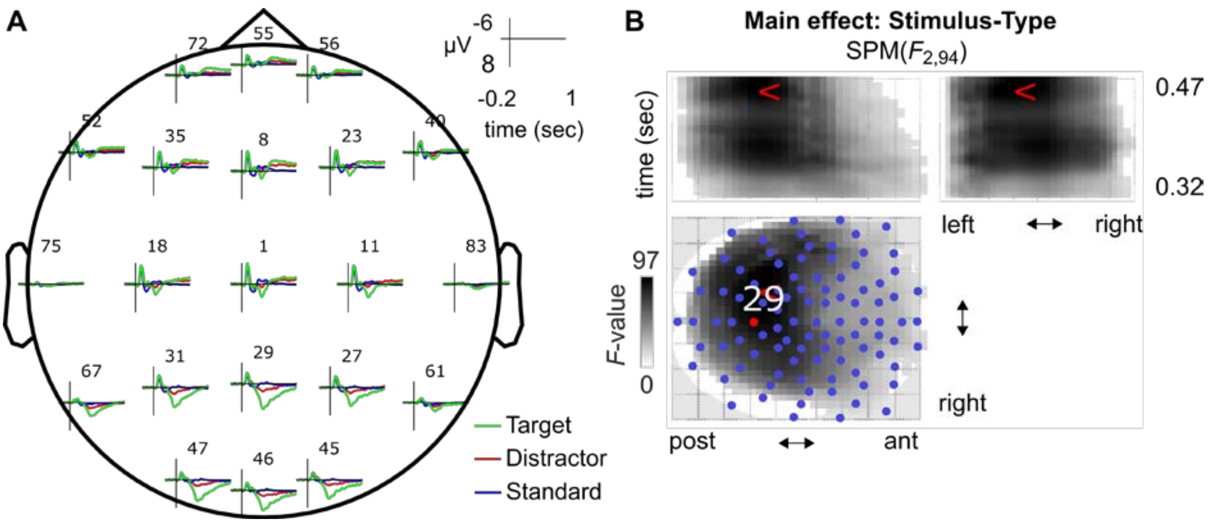

Figure S1-2. (A) Grandaverage waveforms (pooled across subjects and conditions) for target (green), distractor (red), and standard (blue) stimulus type at selected electrode sites (21 out of 96 recorded). Data are referenced to linked mastoids. **(B)** Main effect of Stimulus-Type within P3b window (320−470 ms). A paired-sample *t*-test (within subject) was performed and the results were assessed with F-contrast. The summary statistic scalp-time data volume was thresholded at p <.001 (uncorrected) with FWE correction p <.05 at cluster-level, based on random field theory. Blue dots mark electrode sites and the grey arrows point to the global maximum. For details, see **Table S1-9**.

Table S1-9. Main effect of factor Stimulus-Type within P3b window (320−470 ms). Statistical results of scalp-time analysis.

**Cluster-level**		**Peak-level**				
$p_{FWE\text{-}corr}$	K_E	***F*-value**	$p_{uncorrected}$	**mm**	**mm**	**ms**
<.001	24201	96.49	<.001	−17	−41	456

Statistics: $p_{FWE\text{-}corr}$ = Family-wise-error corrected *p*-values adjusted for search volume. K_E = cluster with *K* elements. Height threshold: $F = 7.33$, $p = .001$ (uncorrected); df = (2, 94); Extent threshold: $k = 54$ bins;

Smoothness FWHM = 35.3 5.1 36.6 mm mm ms; Expected bins per cluster <*k*> = 54.35; Search vol.: 2808136 = 30732 bins = 37.7 resels; Bin size: 4.2 5.4 4.0 mm mm ms; (resel = 709 bins).

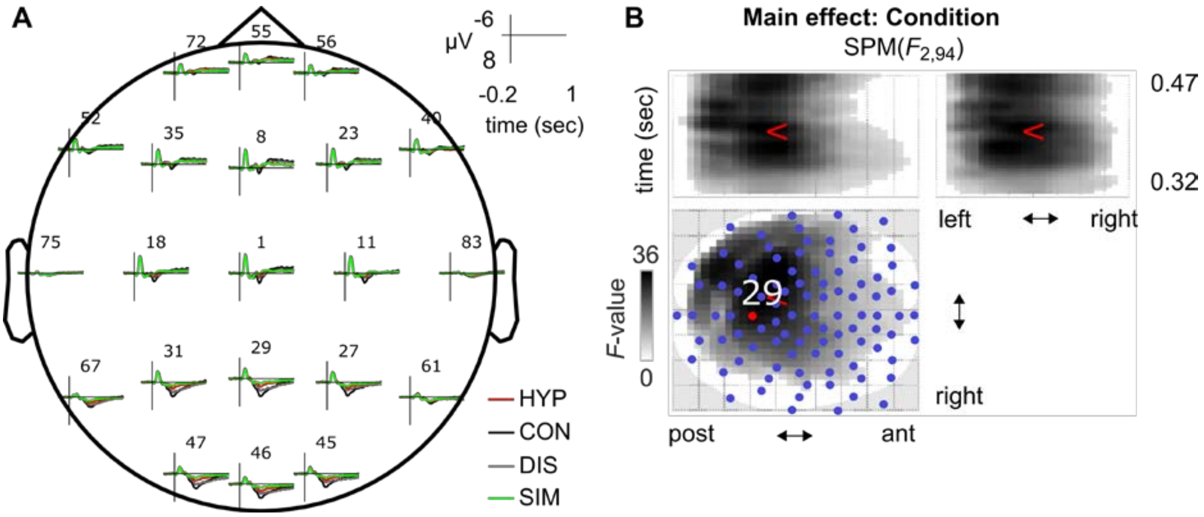

Figure S1-3. (A) Grandaverage waveforms (pooled across subjects and Stimulus-Type) at selected electrode sites (21 out of 64 recorded) in hypnosis (HYP, red), control (CON, black), distraction (DIS, grey), and simulation (SIM, green). Data are referenced to linked mastoids. **(B)** Main effect of Condition (HYP < CON) within P3b window (320−470 ms). A one-sample *t*-test was performed and the results were assessed with a *t*-contrast. The summary statistic scalp-time data volume was thresholded at p <.001 (uncorrected) with FWE correction at cluster-level, p <.05, based on random field theory. Blue dots mark electrode sites and the red arrows point to the global maximum. For details, see **Table S1-10**.

Table S1-10. Main effect of factor Condition. Statistical results of scalp-time analysis.

**Cluster-level**		**Peak-level**				
$P_{FWE\text{-}corr}$	K_E	***F*-value**	$P_{uncorrected}$	**mm**	**mm**	**ms**
<.001	17514	36.1	<.001	−8	−36	400

Statistics: $p_{FWE\text{-}corr}$ = Family-wise-error corrected *p*-values adjusted for search volume. K_E = cluster with *K* elements. Height threshold: $t = 3.23$, $p = .001$ (uncorrected); df = 59; Extent threshold: $k = 49$ bins; Smoothness FWHM = 36.8 49.8 33.7 mm mm ms; Expected bins per cluster, <*k*> = 49.38; Search vol.: 2801649 = 30661 bins = 38.7 resels; Bin size: 4.2 5.4 4.0 mm mm ms; (resel = 677 bins).

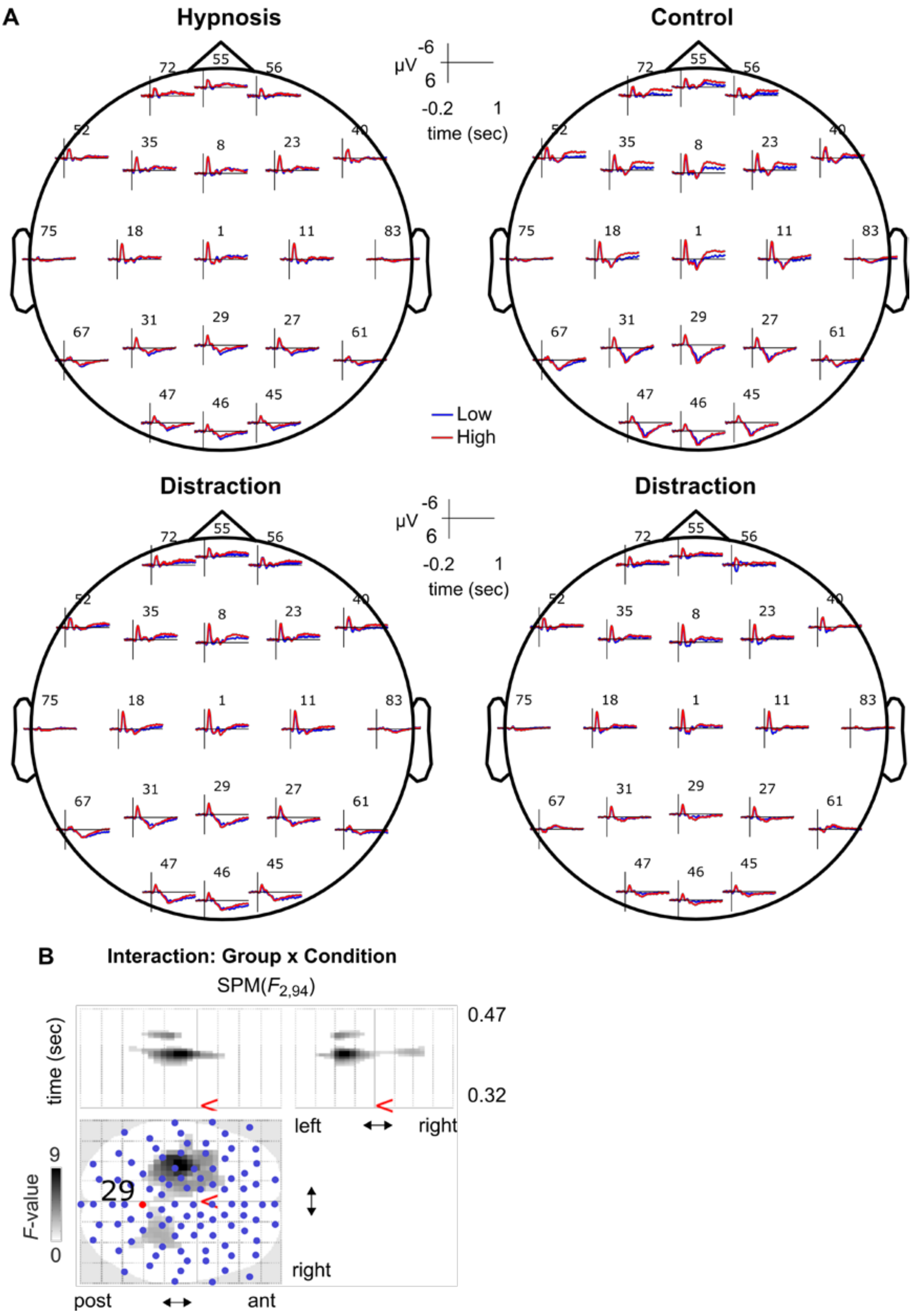

Figure S1-4. (A) Grandaverage waveforms (pooled across Stimulus-Type) for low and high suggestibles at selected electrode sites (21 out of 96 recorded) in hypnosis (HYP, red), control (CON, black), distraction (DIS, grey), and simulation (SIM, green). Data are referenced to linked mastoids. **(B)** Interaction of Susceptibility-Group x Condition within P3b window (320−470 ms). A two-sample *t*-test was performed and the results were assessed with an *F*-contrast. The summary statistic scalp-time data volume was thresholded at $p < .001$ (uncorrected) with FWE correction at cluster-level, $p < .05$, based on random field theory. Blue dots mark electrode sites and the grey arrows point to the global maximum. For details, see **Table S1-11**.

Table S1-11. Interaction of Susceptibility-Group x Condition within P3b window (320−470 ms). Statistical results of scalp-time analysis.

**Cluster-level**		**Peak-level**				
$\boldsymbol{P}_{\text{FWE-corr}}$	$\mathbf{K}_{\mathbf{E}}$	***F*-value**	$\boldsymbol{P}_{\text{uncorrected}}$	**mm**	**mm**	**ms**
<.001	320	9.2	<.001	−26	−14	396

Statistics: $p_{FWE\text{-}corr}$ = Family-wise-error corrected *p*-values adjusted for search volume. K_E = cluster with *K* elements. Height threshold: $F = 5.73$, $p = .001$ (uncorrected); df = 3, 181; Extent threshold: k =48 bins; Smoothness FWHM = 37.2 51.0 31.8 mm mm ms; Expected bins per cluster, $<k>$ = 34.2; Search vol.: 2801649 = 30661 bins = 39.7 resels; Bin size: 4.2 5.4 4.0 mm mm ms; (resel = 660 bins).

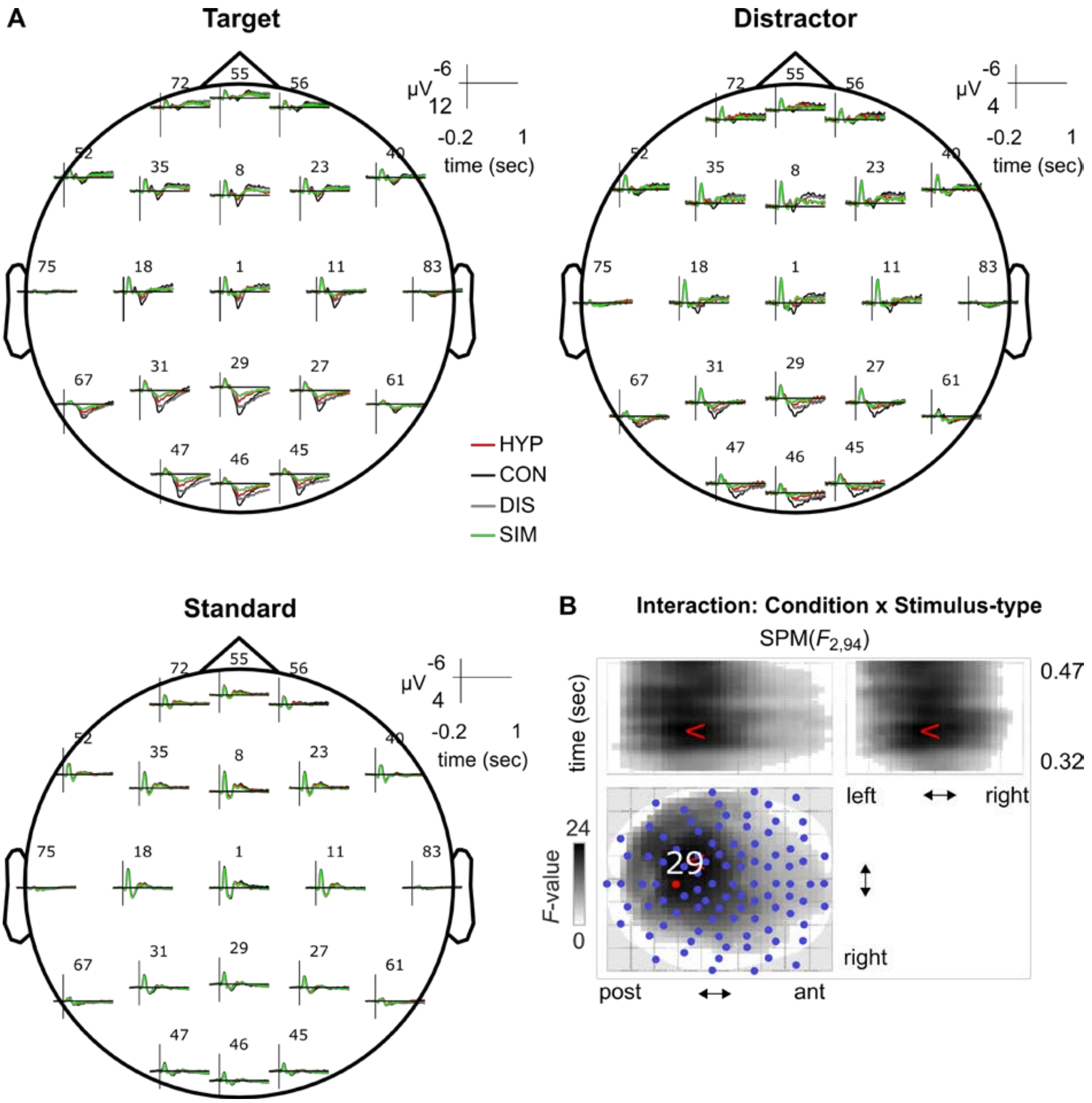

Figure S1-5. (A) Grandaverage waveforms (pooled across subjects) for target, distractor, and standard stimulus at selected electrode sites (21 out of 96 recorded) in hypnosis (HYP, red), control (CON, black), distraction (DIS, grey), and simulation (SIM, green). Data are referenced to linked mastoids. **(B)** Interaction of Condition x Stimulus-Type within P3b window (320−470 ms). A two-sample *t*-test was performed and the results were assessed

with an *F*-contrast. The summary statistic scalp-time data volume was thresholded at p <.001 (uncorrected) with FWE correction at cluster-level, p <.05, based on random field theory. Blue dots mark electrode sites and the grey arrows point to the global maximum. For details, see **Table S1-12**.

Table S1-12. Interaction of Condition x Stimulus-Type within P3b window (320−470 ms). Statistical results of scalp-time analysis.

Cluster-level		Peak-level				
$P_{FWE\text{-}corr}$	K_E	*F*-value	$P_{uncorrected}$	mm	mm	ms
<.001	18380	23.8	<.001	−13	−41	376

Statistics: $p_{FWE\text{-}corr}$ = Family-wise-error corrected *p*-values adjusted for search volume. K_E = cluster with *K* elements. Height threshold: $t = 0$, $p = .001$ (uncorrected); df = 6, 282; Extent threshold: k =34 bins; Smoothness FWHM = 35.6 49.1 26.8 mm mm ms; Expected bins per cluster, <*k*> = 34.2; Search vol.: 2777252 = 30394 bins = 47.8 resels; Bin size: 4.2 5.4 4.0 mm mm ms; (resel = 514 bins).

Table S1-13. Statistical results of the *t*-contrasts for the scalp-time data separated by stimulus type and condition to disentangle the interaction Condition x Stimulus-Type at sensor-level within the P3b window (320–470 ms).

Stimulus	***t*-Contrasts (CON>HYP)**						
	Cluster-level		Peak-level				
	$P_{FWE\text{-}corr}$	K_E	*t-value*	P_{uncorr}	mm	mm	ms
**Target**	<.001	16471	8.21	<.001	−4	−30	356
**Distractor**	<.001	5126	5.16	<.001	−34	−68	408
**Standard**	<.001	3623	5.45	<.001	−38	−9	352
	***t*-Contrasts (DIS>HYP)**						
**Target**	<.001	4383	5.61	<.001	−13	−30	468
**Standard**	<.001	1522	4.66	<.001	−38	−14	356
	***t*-Contrasts (SIM<HYP)**						
**Target**	<.001	3598	4.50	<.001	−51	−25	468
	***t*-Contrasts (SIM>HYP)**						
**Standard**	<.001	1535	4.93	<.001	47	40	324

Statistics: $p_{FWE\text{-}corr}$ = Family-wise-error corrected *p*-values adjusted for search volume. K_E = cluster with *K* elements; Height threshold: $t = 3.12$, $p = .001$ (uncorrected); df = 490; Extent threshold: $k = 76$ bins; Smoothness FWHM = 39.1 52.6 28.8 mm mm ms; Expected bins per cluster, <*k*> = 75.6; Search vol.: 2739788 = 29984 bins = 34.6 resels

SOURCE ANALYSIS

Table S1-14. Statistical results of the one-sample *t*-test in the control (CON). Table shows sources that revealed significant activities during target, standard and distractor processing within the P3b window (320–470 ms).

	CON: Target							
	Cluster-level			Peak-level				
						MNI		
**Region**	p_{Label}	$p_{FWE-corr}$	K_E	*t*-value	p_{uncorr}	mm	mm	mm
r MTG	.53	<.001	109	6.49	<.001	62	−18	−17
l PCu	.48	<.001	160	6.08	<.001	−9	−61	51
r PCu	.38	<.001	132	6.07	<.001	10	−59	54
l MTG	.05	.001	104	5.95	<.001	−47	−13	−15
r PrG	.32	.006	70	5.86	<.001	6	−25	71
r LOC	.40	<.001	118	5.79	<.001	38	−81	−17
l PrG	.25	.012	59	5.70	<.001	−8	−16	72
r MFG	.60	.031	44	5.55	<.001	43	21	44
l SPL	.36	.014	56	5.54	<.001	−38	−40	60
l MFG	.56	.033	43	5.32	<.001	−34	27	43
l FP	.47	.027	46	5.10	<.001	−11	66	0
l OFG	.40	.012	59	4.89	<.001	−22	−78	−7
l ITG	.40	.007	67	4.42	<.001	−55	−62	−12
	**CON: Standard**							
l PrG	.63	.005	94	9.06	<.001	−62	−12	19
l MTG	.45	<.001	521	8.41	<.001	−54	1	−34
r COC	.39	<.001	169	8.32	<.001	56	−14	19
l FP	.11	<.001	156	8.02	<.001	−16	58	13
r MTG	.32	<.001	464	7.78	<.001	54	4	−30
No label		.001	136	7.15	<.001	21	23	34
l SMG	.53	.012	76	6.98	<.001	−60	−50	29
r FP	.54	<.001	181	6.30	<.001	17	64	10
l MFG	.04	<.001	165	5.84	<.001	−25	22	33
r SMC	.35	.036	51	5.67	<.001	9	4	68
r SPL	.37	.028	57	5.32	<.001	20	−53	61
l LOC	.26	.013	73	5.27	<.001	−18	−59	58
l SPL	.36	.031	54	5.10	<.001	−38	−40	60
r PrG	.39	.036	51	4.71	<.001	7	−41	70
	**CON: Distractor**							
l STG	.07	.001	97	7.65	<.001	−47	−9	−16
r MTG	.08	.002	91	7.16	<.001	48	−7	−22
r MFG	.28	.021	53	6.45	<.001	28	28	36
l MFG	.41	.031	46	6.01	<.001	−28	26	49
l SPL	.46	.013	61	5.46	<.001	−17	−56	67
r SPL	.31	.015	58	5.10	<.001	14	−56	68
r OP	.63	.002	96	5.08	<.001	17	−102	−7
r PCG	.29	.025	50	5.00	<.001	12	−18	71

MNI = Montreal Neurological Institute Coordinate System; l = left; r = right; MTG = middle temporal gyrus; SPL = superior parietal lobule; LOC = lateral occipital cortex; OFG = occipital fusiform gyrus; PrG = precentral gyrus; SFG = superior frontal gyrus; SMA = supplementary motor cortex (juxtapostional lobule cortex); p_{Label} = probability of the vertex being a member of the different labelled regions within the Harvard-Oxford Subcortical/Cortical Structural Atlas. K_E = cluster with K_E elements; Statistics: $p_{FWE\text{-}corr}$ = Family-wise-error corrected *p*-values adjusted for search volume. Height threshold: $T = 3.12$, $p = .001$ (uncorrected); df = [1, 94]; HYP: Extent threshold: 45; Smoothness FWHM = 4.0 4.0 4.0 (vertices); Expected bins per cluster, $<k> = 15.6$; Search vol.: 8196 = 112.2 resels (resel = 73.1 vertices). Target: Extent threshold: 43; Smoothness FWHM = 4.3 4.3 4.3 (vertices); Expected bins per cluster, $<k> = 15.6$; Search vol.: 8196 = 103.3 resels (resel = 79.4 vertices). Standard: Extent threshold: 51; Smoothness FWHM = 4.8 4.8 4.8 (vertices); Expected bins per cluster, $<k> = 21.8$; Search vol.: 8196 = 73.6 resels (resel = 111.4 vertices). Distractor: Extent threshold: 46; Smoothness FWHM = 4.4 4.4 4.4 (vertices); Expected bins per cluster, $<k> = 16.8$; Search vol.: 8196 = 96.2 resels (resel = 85.2 vertices).

Table S1-15. Statistical results of the *t*-contrast: Target > Standard in the control (CON), distraction (DIS), hypnosis (HYP), and simulation (SIM) condition at the source-level within the P3b window (320–470 ms).

	CON: Target > Standard							
	Cluster-level			Peak-level				
						MNI		
**Region**	p_{Label}	$p_{FWE\text{-}corr}$	K_E	*t*-value	p_{uncorr}	**mm**	**mm**	**mm**
l LOC	.37	<.001	235	7.75	<.001	−10	−62	62
r PCu	.42	<.001	154	7.67	<.001	8	−61	60
r MTG	.53	.001	83	7.35	<.001	62	−18	−17
l MTG	.05	.002	78	6.79	<.001	−47	−13	−15
r PrG	.29	.001	85	6.39	<.001	12	−18	71
r OFG	.40	<.001	132	5.98	<.001	38	−81	−17
l OFG	.40	<.001	125	5.85	<.001	−22	−78	−7
l PrG	.21	.002	77	5.64	<.001	−10	−17	70
l LOC	.73	.045	35	5.32	<.001	−43	−73	36
l MFG	.45	.029	41	4.58	<.001	−42	21	37
l ITG	.50	.003	72	4.19	<.001	−57	−60	−17
	**DIS: Target > Standard**							
l LOC	.39	<.001	265	8.82	<.001	−12	−59	65
r PCu	.32	<.001	291	8.66	<.001	11	−57	58
r OP	.51	.001	97	5.96	<.001	20	−99	−12
l PrG	.38	<.001	117	5.75	<.001	−14	−27	70
r MTG	.46	<.001	117	5.74	<.001	53	−1	−29
l OP	.53	<.001	244	5.32	<.001	−11	−94	−6
l MTG	.05	.006	63	4.86	<.001	−47	−13	−15
l FP	.44	.007	61	4.51	<.001	−16	62	10
r FP	.34	.050	34	4.13	<.001	14	64	−4
	**HYP: Target > Standard**							
l MTG	.50	.005	63	6.59	<.001	−52	0	−29
r MTG	.30	<.001	103	6.57	<.001	58	−14	−19
r OFG	.59	<.001	102	5.81	<.001	19	−84	−14
l OFG	.40	.009	54	5.78	<.001	−22	−78	−7
r LOC	.55	.016	47	5.39	<.001	20	−83	42
l LOC	.47	<.001	138	5.12	<.001	−15	−84	40
r PCu	.38	.002	77	4.94	<.001	10	−59	54
l SFG	.32	.019	45	4.21	<.001	−10	3	66
r SFG	.35	.003	70	4.00	<.001	9	4	68
	**SIM: Target > Standard**							
r MTG	.46	<.001	113	5.99	<.001	53	−1	−29
l MTG	.50	.003	68	5.76	<.001	−52	0	−29
r PCu	.24	.007	57	5.30	<.001	13	−55	57
l LOC	.22	.004	65	5.24	<.001	−13	−59	58
r OP	.31	.036	36	4.87	<.001	17	−94	−15
r PrG	.29	.013	49	4.71	<.001	10	−23	71
l SMC	.34	.046	33	4.13	<.001	−6	−10	71
l MFG	.45	.036	36	4.04	<.001	−42	21	37

MNI = Montreal Neurological Institute Coordinate System; l = left; r = right; LOC = lateral occipital cortex; PCu = precuneous; MTG = middle temporal gyrus; OFG = occipital fusiform gyrus; PrG = precentral gyrus; SFG = superior frontal gyrus; SMA = supplementary motor cortex (juxtapostional lobule cortex); p_{Label} = probability of the vertex being a member of the different labelled regions within the Harvard-Oxford Subcortical/Cortical Structural Atlas. K_E = cluster with K_E elements; Statistics: $p_{FWE\text{-}corr}$ = Family-wise-error corrected *p*-values adjusted for search volume. Height threshold: $T = 3.12$, $p = .001$ (uncorrected); df = [1, 94]; HYP: Extent threshold: 45; Smoothness FWHM = 4.0 4.0 4.0 (vertices); Expected bins per cluster, <*k*> = 15.6; Search vol.: 8196 = 112.2 resels (resel = 73.1 vertices). CON: Extent threshold: 35; Smoothness FWHM = 4.0 4.0 4.0 (vertices); Expected bins per cluster, <*k*> = 15.6; Search vol.: 8196 = 125.7 resels (resel = 65.2 vertices). DIS: df = [1, 225]; Extent threshold: 36; Smoothness FWHM = 4.1 4.1 4.1 (vertices); Expected bins per cluster, <*k*> = 15.1; Search vol.: 8196 = 115.4 resels (resel = 71.0 vertices). SIM: df = [1, 234]; Extent threshold: 37; Smoothness FWHM = 4.2 4.2 4.2 (vertices); Expected bins per cluster, <*k*> = 15.3; Search vol.: 8196 = 114.2 resels (resel = 71.8 vertices).

Table S1-16. Statistical results of the *t*-contrast: Target > Distractor in the hypnosis (HYP), control (CON), distraction (DIS), and simulation (SIM) condition at the source-level within the P3b window (320–470 ms).

	CON: Target > Distractor							
	Cluster-level			Peak-level				
						MNI		
Region	p_{Label}	$p_{FWE\text{-}corr}$	K_E	*t*-value	p_{uncorr}	mm	mm	mm
l LOC	.37	<.001	154	6.63	<.001	−10	−62	62
r PCu	.42	<.001	129	6.39	<.001	8	−61	60
r MTG	.22	.005	64	5.91	<.001	55	−17	−17
l PrG	.31	.011	54	5.63	<.001	−37	−34	44
l MTG	.36	.007	60	5.40	<.001	−57	−14	−20
r PRG	.29	.006	62	5.15	<.001	12	−18	71
l OFG	.46	.036	38	4.83	<.001	−21	−74	−8
r OFG	.53	.006	62	4.74	<.001	19	−76	−13
l PRG	.15	.031	40	4.40	<.001	−10	−12	69
	DIS: Target > Distractor							
l LOC	.39	<.001	224	7.52	<.001	−12	−59	65
r PCu	.32	.001	90	7.22	<.001	11	−57	58
r PrG	.36	.002	77	5.93	<.001	14	−20	69
l PrG	.38	.008	60	4.49	<.001	−14	−27	70
l OP	.48	.047	35	3.88	<.001	0	−96	−4
	HYP: Target > Distractor							
l OFG	.40	.038	36	4.56	<.001	−22	−78	−7
r MTG	.75	.016	47	4.07	<.001	65	−15	−17
r PCu	.38	.008	56	4.03	<.001	10	−59	54
l MTG	.28	.033	38	3.99	<.001	−59	−12	−24
l PCu	.48	.004	66	3.97	<.001	−7	−63	58
	SIM: Target > Distractor							
		n.s.			n.s.			

MNI = Montreal Neurological Institute Coordinate System; l = left; r = right; MTG = middle temporal gyrus; SPL = superior parietal lobule; LOC = lateral occipital cortex; OFG = occipital fusiform gyrus; PRG = precentral gyrus; SFG = superior frontal gyrus; SMA = supplementary motor cortex (juxtapostional lobule cortex); p_{Label} = probability of the vertex being a member of the different labelled regions within the Harvard-Oxford Subcortical/Cortical Structural Atlas. K_E = cluster with K_E elements; Statistics: $p_{FWE\text{-}corr}$ = Family-wise-error corrected *p*-values adjusted for search volume. Height threshold: $T = 3.12$, $p = .001$ (uncorrected); df = [1, 94]; HYP: Extent threshold: 36; Smoothness FWHM = 4.0 4.0 4.0 (vertices); Expected bins per cluster, $<k> = 15.6$; Search vol.: 8196 = 132.5 resels (resel = 61.8 vertices). CON: Extent threshold: 35; Smoothness FWHM = 4.0 4.0 4.0 (vertices); Expected bins per cluster, $<k> = 15.6$; Search vol.: 8196 = 125.7 resels (resel = 65.2 vertices). DIS: Extent threshold: 36; Smoothness FWHM = 4.1 4.1 4.1 (vertices); Expected bins per cluster, $<k> = 15.1$; Search vol.: 8196 = 122.3 resels (resel = 67.0 vertices).

Table S1-17. Statistics of the effect of distraction (CON > DIS), hypnosis (CON > HYP), and simulation (CON > SIM) for the processing of target stimuli at source-level.

	Target: CON > DIS							
	Cluster-level			Peak-level				
						MNI		
Region	p_{Label}	$p_{FWE\text{-}corr}$	K_E	*t*-value	p_{uncorr}	mm	mm	mm
l LOC	.18	.003	79	5.77	<.001	−18	−59	50
r SPL	.24	.003	77	5.23	<.001	18	−56	53
	**Target: CON > HYP**							
l SPL	.36	<.001	188	6.26	<.001	−38	−40	60
r SPL	.31	.003	79	5.58	<.001	21	−55	65
	**Target: CON > SIM**							
l LOC	.22	<.001	226	9.62	<.001	−13	−59	58
r PCu	.32	<.001	143	9.41	<.001	11	−57	58
r PrG	.34	.001	96	6.52	<.001	15	−26	67
r OFG	.40	.001	89	6.07	<.001	38	−81	−17
l PrG	.38	.001	83	4.93	<.001	−14	−27	70

HYP = hypnosis; CON = control, DIS = distraction; SIM = simulation; l = left; r = right; LOC = lateral occipital cortex; SPL = superior parietal cortex; OFG = occipital fusiform gyrus; PCu = precuneous. RMS = root mean square (a.u.); MNI = Montreal Neurological Institute Coordinate System; $p_{FWE\text{-}corr}$ = Family-wise-error corrected *p*-values adjusted for search volume; K_E = cluster with *K* elements; Height threshold: $T = 3.13$, $p = .001$ (uncorrected); DIS: df = 234; Extent threshold: 77; Smoothness FWHM = 4.2 4.2 4.2 (vertices); Expected bins per cluster, $<k> = 15.2$; Search vol.: 8196 = 114.5 resels (resel = 71.6 vertices); HYP: df = 235; Extent threshold: 79; Smoothness FWHM = 4.2 4.2 4.2 (vertices); Expected bins per cluster, $<k> = 15.6$; Search vol.: 8196 = 112.2 resels (resel = 73.1 vertices); SIM: df = 232; Extent threshold: 66; Smoothness FWHM = 4.2 4.2 4.2 (vertices); Expected bins per cluster, $<k> = 13.6$; Search vol.: 8196 = 128.1 resels (resel = 64.0 vertices);

REFERENCES

SPM. (2018). Group Analysis. Retrieved from https://en.wikibooks.org/w/index.php?title=SPM/Group_Analysis&oldid=3373190
