# Supplementary material for: Suggested deafness during hypnosis and simulation of hypnosis compared to a distraction and control condition: A study on subjective experience and cortical brain responses
Source: PLoS One. 2020 Oct 29;15(10):e0240832. doi: 10.1371/journal.pone.0240832 (PMC7595429; doi:10.1371/journal.pone.0240832)
Supplement: S2 File — (PDF) [file pone.0240832.s002.pdf]

Supplemental information 2 to the article:

Suggested deafness during hypnosis and simulation of hypnosis compared to a distraction and control condition: A study on subjective experience and cortical brain responses.

Marcel Franz[1], Barbara Schmidt[1], Holger Hecht[1], Ewald Naumann[2] and Wolfgang H.R. Miltner[1,3]

[1]Department of Clinical Psychology, Friedrich Schiller University Jena, Jena, Germany

[2]Institute of Psychology, University of Trier, Trier, Germany

[3]Medical School of Friedrich Schiller University Jena, Jena, Germany

The following text is a corresponding translation of the original German instructions.

S2-1 Control instruction (CON)

You will hear different tones in this condition. In the first section you will hear three different tones: a lower, a middle, and a higher tone. It is your task to respond to the higher tone by pressing the button on your right armrest with your right hand. In the second section, you hear a different tone to which you should not respond. In between, I will ask how you felt the tones.

S2-2 Disattention Instruction (DIS)

In the following section of our study you will see a film. I would like to ask you to watch this film very closely and to try to remember as many details as possible. This is important because you will be asked to answer a few questions about the film afterwards in order to receive a reward for every correct answer. The reward is 50 cents for each question you answer correctly. Therefore, please watch the film as carefully as possible and try to remember as many details as possible so that you can answer the questions later in the best possible way.
You will hear different tones during the film. In the first section, you will hear three different tones: a lower, a middle, and a higher tone. It is your task to respond to the higher tone by pressing the button on your right armrest with your right hand. In the second section, you hear a different tone to which you should not respond. In between, I will ask how you felt the tones.

S2-3 Hypnosis condition (HYP)

Rest 1
[EEG REST MEASUREMENT WITH OPEN EYES FOR 2 MINUTES]

Can you understand me well through the earphones? (Waiting for a response)

Hypnosis Introduction

Now, we start our hypnosis session. I want you to relax as best as possible and to sit comfortably as possible in the chair for the next time.
Much of what you will experience in the following, you already know from the hypnosis group test in which you participated. Today, we want to examine how you and your brain will respond

to further hypnotic suggestions. Of course, nothing will happen here that is not in line with your own will or that could expose you in any way.

Just watch what happens and try to allow all the experiences that I will suggest to you. Do not push yourself to do anything that does not work, but try not to hold back things that do work. You can do absolutely nothing wrong. Just be completely relaxed.
Do you see the camera above the glass surface in front of you? I will call it “sign”. I would ask you to relax in your chair, keep your eyes on the sign, and listen to my voice. In the meantime, I will give you some instructions that will help you to relax and gradually enter a hypnotic state. Please keep your eyes on the sign and keep paying attention to my words as you look at it. You can only enter a hypnotic state if you want to. Try your best to focus on the sign and pay close attention to my words. If your thoughts wander, just bring your thoughts back to the sign and my words. Notice how the sign changes, how it may sometimes become unclear, sometimes clearer. Whatever is going through your mind, allow it, but keep fixing the sign.

Relaxation under hypnosis is very similar to the state just before falling asleep. But you will not sleep in the usual sense because you will continue to hear my voice and will be able to focus your thoughts on what I am telling you.
You are pleasantly relaxed, but you will relax much more, much more.
Just pay close attention to my voice. Sometimes my voice may seem to change or sound as if it is far away. That's fine. If you start to feel sleepy, that's fine too. Accept whatever happens and just keep listening to my voice as you relax more and more.

As you imagine the relaxation, your muscles will relax. Start with your right foot, relax the muscles of your right leg ... now the muscles of your left leg ... relax completely. Relax your right hand, your forearm, your upper arm and your shoulder ... now your left hand ... and your forearm ... and your upper arm ... and shoulder ... relax your neck and your chest ... completely relaxed.
As you relax more and more, your body will feel heavy or maybe numb. You will begin to feel numbness or heaviness in your legs and feet ... in your hands and arms ... in your whole body ... as if you are sinking deeper into your chair. The chair is stable, it will hold your body as it feels heavier and heavier.
Your eyelids feel heavy, very heavy. You start to feel relaxed and sleepy. Your eyes burn a little and your eyelids feel very heavy. Your eyelids are getting heavier ...

Your eyes get blurry from the effort. You can hardly see the sign anymore, your eyes are so strained. Soon you won't be able to keep your eyes open. Your eyelids are heavy. Very heavy. Getting heavier and heavier. They push down, deeper and deeper. There seem to be weights on your eyelids pushing them deeper and deeper…. Your eyes are flickering ... flickering ... closing, closing.

Your eyes are now closed. Just keep your eyes closed until I ask you to open them.
You are relaxed, very relaxed. You can relax even more if you just let yourself go. You can reach a state of even deeper, complete relaxation. You become increasingly sleepy. You feel a pleasant feeling of numbness and heaviness throughout your body. You begin to feel so relaxed, so sleepy. It is easier to turn your thoughts away from other things and focus your attention only on my voice. Soon you will only listen drowsily to my voice as you relax more and more.
Now I want you to watch your breathing ... Take a deep breath ... deep breath. Then hold your breath ... And then exhale through your mouth. Maybe you can already feel peace and relaxation spreading through your body ... Now breathe in and out again calmly and evenly - in and out ... As you breathe in, your abdominal wall bulges slightly upwards, and as you breathe out, it falls

slightly downwards again ... in ... and out ... in ... and out. How this inhalation and exhalation goes hand in hand with peace and relaxation! If you continue to breathe calmly, sooner or later you will find yourself in a very pleasant inner peace without having to do anything for it ... You breathe in and out ... in and out. Whenever you breathe in, you take in oxygen and energy ... And every time you breathe out, you relax more deeply and can let go ... With every breath you can let go more ... with every single breath you take ... You exhale and let go of everything that disturbs you ... you exhale and inner peace will come more and more - all by itself ... and with every single breath the relaxation becomes deeper and deeper ... as deep as it is possible for you now.

All you have to do is pay attention to your breathing and feel how your breath flows in and out ... in and out ... And you can imagine how the air you breathe flows through your nose through your windpipe to your lungs and back again...
Some people focus their attention on the muscles of the body and feel how they relax more and more deeply with each breath, as if breathing tells the muscles to relax even further ... You don't need to do anything at all ... just feel and notice. Often you can feel the relaxation first in the temporal region. It moves from the forehead to the eyes ... to the root of the nose via the cheeks and the nostrils ... over the lips and into the mouth.
And this relaxation also spreads to the neck, because with every breath the muscles there also become more relaxed and looser ... You don't have to do anything. Just feel and notice how the relaxation flows - like a stream - over your shoulders, down your back ... Every time you breathe out you can feel the relaxation - also in the upper arms ... in your forearms ... and finally the fingers join in, the palm of your hand ... right down to your fingertips ... And when you exhale, the air flows gently and smoothly back out through your nose ... Your arms can relax - by themselves ... With every breath you take, peace and relaxation become more and more ... If you pay attention to your nose, you can feel the air flowing in and out through your nose ... And you can feel how gradually the whole head area, the upper part of the body and your arms and hands are seized by a deep feeling of relaxation and calmness ... When you breathe in, you can have the feeling of becoming lighter ... This is quite normal ... You may have noticed that exhaling takes a little longer than inhaling ... With every breath you sink deeper ... and deeper ... and deeper.

You are relaxed, very relaxed. Your whole body feels heavy and relaxed. You feel a pleasantly warm feeling throughout your body as you become more and more sleepy. Sleepy. Very sleepy. Keep your thoughts on what I'm saying; listen to my voice. Soon you will think of nothing but my voice and the words I say as you relax more and more. There is nothing to worry about now. Nothing except what my voice tells you seems important, nothing else is important now. Even my voice may sound a little strange, as if it is coming from a dream, as you sink deeper into this numbness, this heaviness of deep relaxation. Relax completely ...
I will soon start counting from 1 to 10. As I count, you will notice how you fall deeper and deeper into a restful sleep. But you will still be able to do all the things I ask you to do without waking up ...
1… you start to relax even deeper… 2…. Deep, deep into a deep healthy sleep ... 3, 4 ... sleeping deeper and deeper ... 5, 6, 7, ... You sink into a deep, deep sleep. Nothing will disturb you ... Please direct your thoughts to my voice and to the things I am telling you. You will experience many of the things I describe to you ... 8, 9, 10 ... deep asleep. You won't wake up until I ask you to.

Now I want you to realize that you will be able to speak, move, and even open your eyes when I ask you to while you remain in the hypnotic state you are in now. Whatever you do, you will remain hypnotic until I tell you otherwise ...

Verification

Now please stretch out your right arm at shoulder height, palm up. [Wait until participant stretches out his/her arm. If not, repeat the instruction]. Just like that. Please pay close attention to your right hand, how it feels, what is going on inside it. Notice if it feels a little numb or not, or maybe tingling. Now please imagine that you are holding something heavy in your hand, maybe a heavy ball or a billiard ball ... something heavy. Form your fingers around it as if you were holding this heavy object you imagine in your hand.
Now your hand and arm feel heavy as if a weight is pushing them down ... hand and arm start to move down ... as if they are being pushed down ... they are sinking ... sinking ... deeper and deeper ... The arm is getting tired and strained ... deeper ... slowly but surely ... down, down ... the weight is so big, the hand is so heavy ... you feel the weight more and more ... the arm is too heavy to hold it back ... it moves down, down ... deeper and deeper ...
Good. Just return your hand to its original resting position and relax. Whatever you do, you will remain in a hypnotic state until I tell you otherwise ...
Now please try to open your eyes slowly. Very slowly. [Wait until participant is opening his/her eyes. If not, repeat the instruction]. There you go. Please keep your eyes open. I will be silent for the next few minutes as you dive deeper into the relaxed state of hypnosis. Use the time to relax even more and dive deeper into the pleasant state of hypnosis.

Rest 2
[EEG REST MEASUREMENT WITH OPEN EYES FOR 2 MINUTES]

Deafness Induction

I will soon play three different tones for you: a lower, a middle and a higher tone. I want to ask you to press the button on your armrest with your right hand when you hear the higher tone. To do this, please place your right arm on the armrest so that you can easily reach the button. Just show me with your keystroke when you have heard the higher tone. This is your task: press the button when you hear the higher tone. After a short pause, I will play other tones for you. You should just listen to them, nothing else. Just listen. But before that I will give you further hypnotic suggestions. Just pay attention to what is happening and try to allow the experiences that I am about to suggest to you.
I would like to try something with you. I want you to try to block out the tones in your ears. Try not to perceive the tones you hear. Fade out the tones so that you won't hear them for the next time. You can still follow my voice, which lets you immerse yourself completely in hypnosis. My voice will be with you all the time, you can always hear it. It will help you go deeper into trance and forget about all other tones. Your hearing will be switched off from your consciousness for a while and you just forget it. But you can always hear my voice. In fact, it is possible that you can fade out any tones while hypnotized and continue to hear my voice. Just concentrate on my relaxing voice and all other tones then gradually disappear from your consciousness. It is even possible that the tones will lead you even deeper into the trance. Every tone ... even deeper ...

Just imagine that you have cotton wool in your ears. Cotton and wax, so that no tone except my voice can get through to you. You have already tested the earplugs before and you know how strongly they block tones from outside. Remember this sensation and imagine how cotton and wax become more and more dense in your ears and make you deaf and insensitive to noise. Then you know that your ears are protected by the earplugs, cotton wool and wax keep all tones away from you. You can be completely calm and trust my voice that guides you. You can

simply fade out your sense of hearing from your consciousness and forget it for a few minutes. Just blank it out. Exactly, very good ...
Perhaps you are now more aware of the sounds from within, like your breathing sounds or your heartbeat. Or maybe you imagine holding a seashell up to your ear on the beach. You may hear the sound of the sea in your ears. You can also imagine a small babbling stream flowing past you. Listen to the splashing. When you hear it, be happy about it and let it relax you further.
When tones come out of the earphones again later, you will not even notice it. Because cotton and wax protect your hearing, the tones become completely indifferent to you. Everything you hear becomes a single carpet of sound, where everything is flowing together in a great, pleasant murmur. You only hear my voice. The noise from all other sounds will make you sink even deeper into the pleasant trance state. The deafness envelops your hearing like a protective shield and does not allow a single sound to penetrate. Your ears are comfortably deaf. You simply forget your sense of hearing. It is still there, but it no longer concerns your consciousness for a while, so that your mind can relax completely. Recover from all the noises and sounds that reach it every day. You can only hear my relaxing voice.

You are ready to no longer notice anything that comes from outside, except my voice. You may be wondering how you did it ... that you were able to fade out your sense of hearing from your consciousness on your own command. But because you really want it, it is very simple. Your ears are deaf, completely deaf except for my voice. You can enjoy realizing that you have succeeded to put a very pleasant deafness on your hearing by the power of your thoughts. You have managed to leave your hearing more and more to the protection of the earplugs. You can now fully immerse yourself in this deafness. A pleasant deafness - you are now completely deaf.

I will start counting from 1 to 10 and then your sense of hearing will have completely disappeared from your consciousness. With every number I call, your relaxation doubles and you put yourself deeper into the trance.
1 ... always, always your ears become more and more deaf ... 2 ... you sink into twice as deep relaxation and deafness 3 ... 4 ... and you sink once again 5 ... 6 ... your ears are completely protected and deaf 7 ... 8 ... 9 ... 10 ...

[AUDITORY ODDBALL PARADIGM: 500 STIMULI IN TOTAL]

[AFTER 100 STIMULI] Reinforcement 1:

You can still feel how cotton and wax fill your ears with a pleasant numbness. My voice helps you to dive deeper into hypnosis once again. All sounds from outside don't matter to you except my voice.

[AFTER 200 STIMULI] Reinforcement 2:

Keep enjoying the pleasant numbness in your ears. Just listen to my voice and feel your ears relax behind the protection of the earplugs.

[AFTER 300 STIMULI] Reinforcement 3:

You can let yourself fall completely into the state of hypnosis. By listening to my voice, you dive deeper into hypnosis. You are indifferent to all sounds except my voice.

[AFTER 400 STIMULI] Reinforcement 4:

How pleasant the deafness in your ears is! You enjoy that your ears are shielded from everything and let my voice lead you deeper into hypnosis.

[END ODDBALL]

You still feel the cotton and wax in your ears as they lie comfortably in your ears. You are relaxed and your ears are protected. In the following I will play tones that you don't care about. You can fully rely on the earplugs in your ears, which give you a pleasant silence.

[AUDITIVE STEADY STATE PARADIGM: 100 TONES AT 40 Hz]

I ask you to immerse yourself in hypnosis for a few more minutes while you hear nothing from me. Just relax and enjoy the pleasant state you are in. Always keep your eyes open.

Rest 3

[EEG REST MEASUREMENT WITH OPEN EYES FOR 2 MINUTES]

Terminate and Remove hypnosis
Now imagine how you take the wax and cotton wool out of your ears. Your hearing gradually returns to your consciousness and the feeling of numbness subsides. The earplugs in your ears disappear and your ears can hear all the sounds from outside again. Let your hearing slowly return to your consciousness.
When I count down from 10 now, you will hear more and more clearly, the feeling of numbing earplugs on your ears is gradually disappearing. You start hearing clearly again. When I reach 1, you will hear clearly again. You will also feel how your relaxation turns into a pleasant feeling of being rested and you will feel fresh and lively at the end. Good, I start counting now.
10 ... 9 ... 8 ... 7 ... 6 ... 5 ... 4 ... 3 ... 2 ... 1
Your hearing has completely returned to your consciousness. Great. Stay relaxed and listen to me. Enjoy these moments a little longer. A feeling of freshness in your lungs accompanies your breathing and you feel increasingly relaxed. I want you to consciously take a few more breaths and take a deep breath through your nose and exhale audibly through your mouth. Feel how you get livelier and fresher with every breath; how confidence and new energy rise within you. When you are at the penultimate of these breaths, you can tighten your muscles and loll around and you are back in the here and now.

S2-4 Simulation instruction (SIM)

We now come to a new section of the experiment. In this section, you should pretend to be hypnotized. You have already had experience with hypnosis, for example at a group session. You will surely remember how you were hypnotized with others as part of our hypnosis study. These experiences can be very useful if you try to act exactly as if you are really hypnotized. You can remember what it was like when you were actually hypnotized the last time and use this as a guide. This may sound difficult, but we ask you to give it a try.
As you try to act as if you are hypnotized in the chamber, we will be watching you on a camera. My colleague will evaluate how well you can pretend to be hypnotized. At the end of this study, when we have collected all the data, the test person who most convincingly pretended to be hypnotized receives a voucher for 30 euros, the second best 20 euros and the third best 10 euros. It is worth the effort!

It is very important that you pretend to be hypnotized throughout the condition. You will hear different tones in this condition. In the first section you will hear three different tones: a lower, a middle and a higher tone. It is your task to respond to the higher tone by pressing a button with your right hand, using the button on your right armrest. In the second section, you will hear a different tone to which you should not react. Throughout the condition, you should pretend to be hypnotized and not to hear all the tones. I'm sure you can do it. In between I will ask how you felt the tones. Then please tell me how you really felt about the tones. You should not simulate the ratings of the tones. But during the presentation of the tones I ask you to pretend to be hypnotized as credibly as possible.

Reinforcement 1:
Keep trying to simulate a hypnotic state as convincingly as possible. Convince the observer that you are really hypnotized and cannot hear anything.

Reinforcement 2:
Remember to convince the observer that you are in a hypnotic state and cannot hear anything. Do your best to give the impression that you are really hypnotized.

Reinforcement 3:
The observer continues to evaluate how well you are pretending to be hypnotized. Try to simulate a hypnotic state as convincingly as possible and pretend you don't hear anything.

Reinforcement 4:
Think of your task to pretend to be hypnotized and not hearing anything. The observer evaluates how well you are able to pretend to be hypnotized.

Test Instructions

Hypnosis Score

How well did you manage to actually hide the stimuli in hypnosis based on the suggestion "You can't hear anything anymore"?

5 = very good (I did not hear any tone anymore)
4 = good (I did not hear most of the tones or I have only perceived the tones in greatly reduced intensity)
3 = a little (I did not hear a few tones anymore or I only perceived the tones with reduced intensity)
2 = hardly (I still heard almost all the tones or I perceived the tones with only a slightly reduced intensity)
1 = not at all (I heard all tones with undiminished intensity)

Simulation Score

Eye movements / blinking:

0 = Eye movements / blinking were frequent and clearly visible
1 = Eye movements / blinking were observed regularly
2 = There was almost no visible eye movement / blinking

Swallowing movements

0 = Swallowing movements were frequent and clearly recognizable
1 = Swallowing movements were regularly observed
2 = There were almost no visible swallowing movements

Pupillary reactions

0 = There were almost no visible pupillary reactions
1 = Pupillary reactions were regularly observed
2 = pupillary reactions were frequent and clearly recognizable

Motor movements (upper body, arms, hands)

0 = movements were frequent and clearly recognizable
1 = Movements were observed regularly
2 = there was almost no visible movement

Distraction Score

1. Which object did the rotating filled garbage bag move at the beginning of the film?
   (correct answer: Car tire)
2. What happened to the foam when it spread on a plate with two burning tea lights?
   (correct answer: The foam burned)
3. What happened to the car tire under which the newspaper was burning?
   (correct answer: Missiles that were attached to the tire ignited and caused the tire to roll)
4. How did the large puddle of oil ignite on the floor?
   (correct answer: Sparks from a firecracker)
5. What color did the sawdust burn?
   (correct answer: Green Blue)
6. How many rotating filled garbage bags could be seen in the entire film?
   (correct answer: Two)
7. Which object hit the filling white balloon?
   (correct answer: Canister)
8. What color was the air mattress in the film?
   (correct answer: Yellow (with green border)
9. Which object was moving on the swinging orange board?
   (correct answer: Filled plastic cup)
10. Was there a shopping cart in the movie?
    (correct answer: No)
